# Seasonal Variability and Effect of Sample Storage on Volatile Components in *Calypogeia azurea*

**DOI:** 10.3390/molecules27082426

**Published:** 2022-04-09

**Authors:** Małgorzata Guzowska, Rafał Wawrzyniak, Wiesław Wasiak

**Affiliations:** Faculty of Chemistry, Adam Mickiewicz University in Poznań, Uniwersytetu Poznańskiego 8, 61-614 Poznań, Poland; rafwawrz@amu.edu.pl (R.W.); wasiakw@amu.edu.pl (W.W.)

**Keywords:** Hepaticae, liverworts, volatile organic compounds, HS-SPME, GC-MS, *Calypogeia azurea*, specialized metabolites

## Abstract

A change in the composition of specialized metabolites is often observed in stressed plants. Phytochemicals play an important role in adapting plants to the environment, particularly overcoming stress conditions such as temperature, humidity, and light intensity. In this study, seasonal variations in the concentrations of volatile organic compounds (VOCs) were analysed in species of *Calypogeia azurea*. The article presents the effect of sample storage on volatile organic compounds present in *Calypogeia* liverwort cells and whether the collection habitats of the sample affect the content of phytochemicals. The VOCs of the species within the liverwort *Calypogeia azurea* were analysed by GC-MS. Compounds were isolated from plant material using the HS-SPME technique. The samples were collected over several years (2019–2021). Of the several dozen samples collected, 79 compounds were isolated, of which 47 were identified.

## 1. Introduction

The chemistry of liverworts (Hepaticae) has been the subject of intensive research in recent decades [1]. Liverworts are the ancestors of all land plants and abundantly produce specialized metabolites, including monoterpenes, sesquiterpenes, monoterpenoids, sesquiterpenoids, and aromatic compounds, many of which exhibit noteworthy biological activities, such as an inhibitory effect on allergic contact dermatitis, cytotoxicity, antibacterial and antifungal activity, anti-insect activity, and antioxidant properties [2,3,4,5].

Due to their morphology, liverworts (Hepaticae) are difficult to classify and identify. Since they are rich in volatile organic compounds, the latter one can be used to access their chemosystematicity [6,7].

Phytochemicals produced by liverworts are unique sources of pharmaceuticals, food additives, flavours, and other industrial materials. The accumulation of these metabolites occurs often in plants subjected to stress, including various stimulators or signal molecules [8]. Modern theories state that the specialized metabolites produced by plants are widely dependent on the environmental condition, duration and intensity of stress, genetic plasticity, and composition of plants. Currently, liverworts are of great biological interest because they contain a wide range of secondary metabolites that are produced to combat a variety of biotics and abiotic stress (microorganisms, insects, UV radiation, and various environments conditions). Most stress factors are seasonal, which means that the total content and relative proportions of specialized metabolites in plants fluctuate with seasonal environmental changes [9]. Consequently, these changes can affect the therapeutic effectiveness of the plant in question.

The genus *Calypogeia raddi* comprises approximately 90 described species distributed throughout the world, with the highest diversity of species observed in the tropics [10,11]. In Holarctis, the species richness of *Calypogeia* is much lower and is represented by only 9–13 species in its different parts [10,12]. In Europe, *Calypogeia* is represented by only eight species: *C. azurea*, *C. integristipula*, *C. neesiana*, *C. suecica*, *C. muelleriana*, *C. sphagnicola*, *C. fissa*, and *C. arguta* [13].

All occur in Poland with the exception of *C. arguta* of the genus *Calypogeia* and are quite common in the flora of the Polish bryophytes, except for *C. fissa* [14]. Most species in the genus *Calypogeia* have colourless and pellucid oil bodies, while coloured oil bodies, including gray to pale brown, purple to blue, are rare [15]. *Calypogeia azurea* is a species with blue oil bodies that has been considered a species of the Holarctic range, green or bluish when alive, yellowish green when dried, from 1.6 to 1.8 mm wide × 7–16 mm long to 2–2.5 mm wide × 7–20 mm long. Leaves are imbricate, ovate to cordate, with maximal width near the base. Blue or deep blue oil bodies under fresh conditions, discernible with a hand lens as dark or blue minute spots, are present in all cells on the top and underside of the leaf [16,17]. The intense blue colour indicates the presence of azulenes, which are not uncommon constituents of essential oils, although in some cases they are presumably formed during sample processing [18,19]. One of these azulenes exhibits anti-inflammatory and anti-ulcer activity [20].

*Calypogeia azurea* prefers to grow on steep walls, for example, stream incisions with acidic loam in coniferous or beech forests. The species sometimes also grows on rotten wood and also in peaty places and north of heathland. Perhaps the future will show that *Calypogeia azurea* is more common than we have to assume now. Searching through relief-rich forests or relief-rich vegetation could lead to new finds. The species can be confused with *C. muelleriana*, which sometimes has a blue tinge. The lower petals of *C. azurea* are more dissected and almost as sharply serrated as those of *C. fissa*. However, *Calypogeia fissa* never has the blue colour of oily bodies [18,19].

Various extraction methods are used to determine the composition of essential oils produced by liverworts. It should be noted that no single analytical technique can provide a complete profile of all volatiles, and it appears that a combination of broad-spectrum profiling methods and targeted methods for the analysis of key volatiles that may be present in very low concentrations will continue to be used.

The basic operation included steps such as prewashing, drying plant materials, freezing, and grinding to obtain a homogeneous sample, and often improving the kinetics of analytic extraction and also increasing the contact of the sample surface with the solvent system. Proper actions must be taken to ensure that potential active constituents are not lost, distorted, or destroyed during the preparation of plant extract [21,22].

The oldest and most basic method is extraction of the sample, in which the analyst aims to separate the analyte of interest from a sample matrix using a solvent with an optimum yield and selectivity [23,24]. Unfortunately, the use of this technique requires purification to remove non-volatile materials that can interfere with subsequent instrumental analysis [25]. For this reason, in this study, it was decided to use the headspace-solid phase microextraction (HS-SPME). This technique is solvent-free and only volatile compounds are analysed, which is particularly advantageous when gas chromatography is used. Compounds were identified by mass spectrometer (MS) using available MS libraries and literature data.

The aim of the study was to demonstrate the effect of abiotic factors on the amount of volatile organic compounds in the oily bodies of liverworts of the species *Calypogeia azurea*. As changes in the external appearance of liverwort leaves were observed over time, the effects of refrigerated and freezer storage of the sample on specialized metabolites were investigated. Taking all the above information into account, studies on the seasonal variability of the chemical composition of liverworts are essential to determine the most productive season for a given species.

## 2. Results and Discussion

### 2.1. Volatiles Present in Calypogeia azurea

Forty-three samples of *Calypogeia azurea* (Table 1) have been analysed for volatile specialized metabolites (Table 2, Table 3, Table 4, Table 5, Table 6, Table 7, Table 8, Table 9 and Table 10).

A total of 79 compounds were detected, of which 47 were identified, accounting for 82.61–92.07% of the total volatile compositions. The remaining compounds are described by means of mass spectra. In terms of content, compounds belonging to sesquiterpenes (61.25–76.54%) and aromatic compounds (1.15–17.69%) dominate. In addition to the above groups of compounds, compounds belonging to sesqiuterpenoids (0.57–6.45%), monoterpenes (0.16–3.39%), monoterpenoids (0.08–1.64%), and aliphatic hydrocarbons (0.09–2.41%) were detected in *C. azurea* cells.

In *Calypogeia azurea*, the dominant compound is 1,4-dimethylazulene (**60**)(9.12–59.23%), responsible for the blue colour of the oil bodies and the 4-(cyclopent-1-enyl)benzoic acid methyl ester (**74**)(1.11–17.31%) responsible for the purple colour of the oil bodies. Methyl-2-methylazulene-1-carboxylate (**79**) is also responsible for the violet colour, but it is present in much smaller amounts (0.01–2.87%).

### 2.2. Storage Effect on Volatile Organic Compounds Present in Calypogeia azurea

One of the goals of the research was to determine the effect of storage of the collected plant material on the composition of volatile organic compounds (VOCs). For this purpose, a sample of liverwort was analysed immediately after cleaning and after storage for 1 and 3 months in a refrigerator (approximately 5 °C) and a freezer (approximately −30 °C).

**Figure 1:**
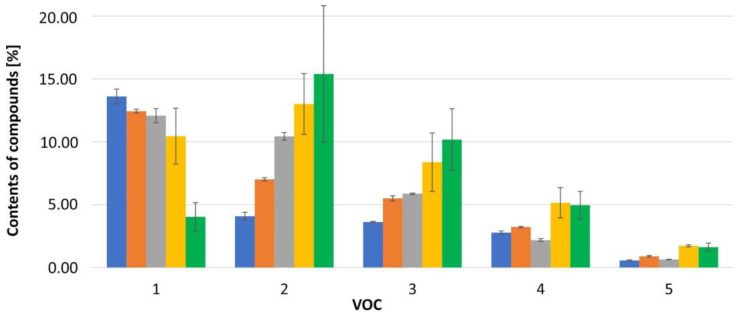
Compare VOC percentages for samples stored refrigerated and frozen for 1 month and 3 months. Compounds: 1: 4-(cyclopent-1-enyl)benzoic acid methyl ester (**74**), 2: bicyclogermacrene (**49**), 3: alloaromadendrene (**40**), 4: germacrene D (**47**), 5: selina-5,11-diene (**41**), and 6: isospathulenol (**67**). Storage: 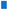 fresh, 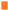 fridge—1 month, 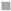 fridge—3 months, 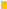 freezer—1 month, 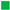 freezer—3 months.

The conditions mentioned above were found to alter the composition of the VOC. The longer the storage time and the lower the temperature, the greater the observed differences in the content of volatile organic compounds. The plant responded to stress by increasing or decreasing the content of specialized metabolites. The highest content drop of 46.10% to 0.42% was recorded for 1,4-dimethylazulene (**60**) and for 4-(cyclopent-1-enyl)benzoic acid methyl ester (**74**) from 13.61% to 4.04%. On the other hand, the largest increase in content was recorded for anastreptene (**25**) from 6.72% to 26.43%, bicyclogermacrene (**49**) from 4.08% to 15.39%, and alloaromadendrene (**40**) from 3.63% to 10.17%. In the case of other compounds, the gains were not so significant. However, in relation to not frozen samples, the samples after freezing had a higher content of such compounds as: isospathulenol (**67**)(1.85%), germacra-4(15),5,10(14)-trien-1-alpha-ol (**77**)(2.89%), and methyl-2-methylazulene-1-carboxylate (**79**)(2.87%). In the frozen samples, maaliol (**59**)(0.25–1.52%) was also detected, which was not present in not frozen cells of *C. azurea*. In the case of other metabolites in the initial storage period, an increase in content was observed, followed by a slight decrease, as exemplified, among others, by selina-5,11-diene (**41**) and germacrene D (**47**). These volatile compounds are present in *Calypogeia azurea* during harvest and decline rapidly in cold stores. The content of limonene (**14**), β-cyclocitral (**19**), and (-)-arystolene (**36**) decreases with the time the sample is stored in the refrigerator and then increases after being placed in the freezer. Temperature and humidity change during storage of *Calypogeia azurea* samples. Mechanical damage may occur during harvesting, transport, and reloading to a storage site; these stresses alter metabolic pathways of plants, which ultimately form the composition of specialized metabolites in stored liverworts. Browning of *Calypogeia azurea* was noticed during sample storage, which may suggest low-temperature injuries, i.e., chilling injury. The defence mechanism of stored plant material depends entirely on types of stress. All of these changes result in certain phenotypic expressions, i.e., colour change, deterioration of compounds, and emission of certain VOCs. The observed changes in the composition of selected VOCs caused by the stress associated with storage in a refrigerator and a freezer are presented in Figure 1.

### 2.3. The Effect of Seasonality on the Content of Volatile Organic Compounds

The liverworts of the *Calypogeia azurea* species are characterized by a visible variation in the composition of specialized metabolites resulting from the vegetation period of the plant. Cyclical changes in the VOC composition were observed in spring, summer, and autumn, repeated in 2019–2021. It is most visible in the example of compounds dominating in the composition of the VOC. The observed changes can be divided into compounds with the highest content in summer. These include: δ-elemene (**22**), anastreptene (**25**), alloaromadendrene (**40**), selina-5,11-diene (**41**), germacrene D (**47**), bicyclogermacrene (**49**), cuparene (**51**), compound IR = 1637 (**63**), and germacra-4(15),5,10(14)-trien-1-alpha-ol (**77**). The second group consists of compounds with the lowest content in summer. They include: compound IR = 902 (**8**), β-pinene (**12**), limonene (**14**), β-cyclocitral (**19**), 1,4-dimethyl-azulene (**60**), and 4-(cyclopent-1-enyl)benzoic acid methyl ester (**74**). A small group of compounds showed a continuous increase or decrease in content from spring to autumn; examples include methyl 2-methylazulene-1-carboxylate (**79**), compound IR = 1224 (**18**), and compound IR = 1392 (**28**). Plants secrete a variety of volatile organic compounds that provide protection against mechanical damage, environmental changes, and pathogens. As a result, these tiny plants developed a unique variety of bioactive compounds as part of their survival strategies. Most sesquiterpenoids increase during spring and peak in summer. These compounds are likely to be elevated during the summer to allow liverworts to cope with abiotic stresses such as high temperatures and droughts.

This percentage drops in autumn, which may be due to *Calypogeia azurea*’s reaction to fewer hours of sunshine per day and the amount of water in the soil. In nature, light plays an irreplaceable role in plant growth and inducing or regulating plant metabolism. In response to light radiation, plants can adapt to changing conditions by releasing and accumulating various specialized metabolites. During experiments, it was shown that plants exposed to drought stress accumulate higher concentrations of specialized metabolites.

**Figure 2:**
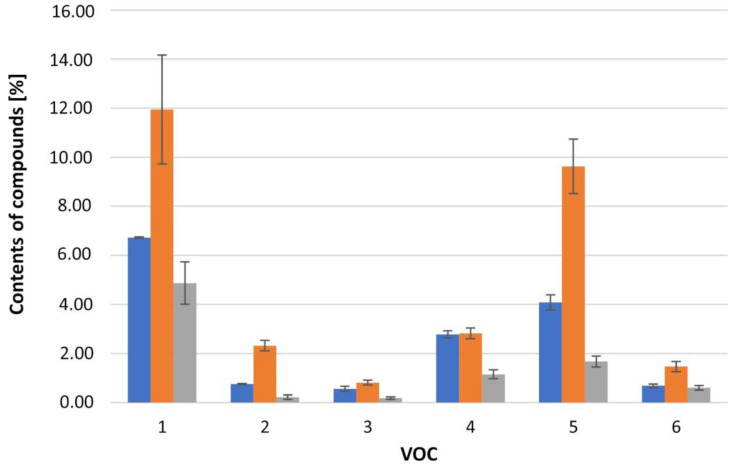
Comparison of the percentage of VOC in the three seasons. Compounds: 1—anastreptene (**25**), 2—δ-elemene (**22**), 3—selina-5,11-diene (**41**), 4—germacrene D (**47**), 5—bicyclogermacrene (**49**), 6—cuparene (**51**). Seasons: 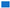 spring, 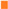 summer, 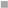 autumn.

The changes in the composition of the specialized metabolites described above are caused by differences in the exposure of the plant to the sun, to water, and nutrients in various stages of vegetation, which in turn affects the metabolic processes of the plant. The described seasonal variability (spring–summer–autumn) for selected compounds is presented in Figure 2. However, no significant differences were observed in the composition of the specialized metabolites resulting from the location of the sites. Samples collected in the Beskid Sądecki or Karkonosze Mountains have a similar composition.

To date, it has been reported in the literature that the accumulation of VOC depends on various environmental factors such as light, temperature, soil water, soil fertility, and salinity, and for most plants, a change in one factor can change the VOC content even if other factors remain constant. External factors can significantly affect some processes related to the growth and development of plants, and even their ability to synthesize specialized metabolites, ultimately leading to changes in general phytochemical profiles that play a strategic role in the production of bioactive substances [26,27,28,29].

### 2.4. Effect of the Type of Substratum on the Content of Secondary Metabolites

On the basis of samples of *C. azurea* grown on decayed wood, it was found that soil influenced the VOC composition. Samples of these plants were istinguished by a much higher content of anastreptene (**25)** and a much lower content of 1,4-dimethyl azulene (**60**) and 4-(cyclopent-1-enyl)benzoic acid methyl ester (**74**) in the spring season. On the other hand, in summer and autumn, the VOC composition was similar to that of *C. azurea* samples growing in forest litter. In samples of *C. azurea* grown in this medium, the presence of compounds not present in liverworts grown in soil was found. They are: 2-methyl-1-propanol (**1**), (Z)-3-hexen-1-ol (**6**), tricyclene (**9**), camphene (**11**), compound IR = 1407 (**30**), (+)-spathulenol (**61**), and compound IR = 1664 (**66**). On the other hand, plants growing on soil had 1,2-dihydro-6-methylnaphthalene (**20**), which was not detected in plants growing on rotting wood. The presented results show that environmental factors related to the substrate, such as temperature, pH, and humidity of the substrate, influence the composition of specialized metabolites produced by liverworts.

**Figure 3:**
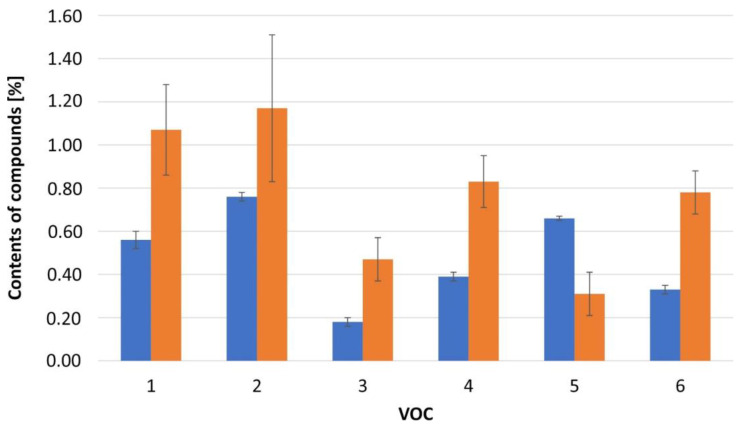
Effect of the place where the sample was collected on the percentage of VOC: Compounds: 1—selina-5,11 -diene (**41**), 2—δ-elemene (**22**), 3—β-elemene (**29**), 4—γ-maaliene (**38**), 5—β-pinene (**12**), 6—α-maaliene (**39**). Collected place: 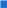 liverworts growing on soil 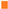 liverworts growing on a rotten tree under the forest litter.

The observed differences in the VOC composition resulting from the substrate, based on the example of selected compounds, are shown in Figure 3.

### 2.5. Volatile Organic Compounds in In Vitro Culture

A separate group of samples was the liverworts collected from the in vitro cultures. Samples were grown from material collected in spring in Beskid Sądecki. The composition of the VOCs was tested after 6 months of cultivation. Each time, the in vitro samples had a composition similar to that of the samples collected in summer in the natural environment. This indicates that in vitro culture was conducted under optimal growth conditions.

### 2.6. Statistical Analysis of the Obtained Results

#### 2.6.1. Observation of Changes Depending on the Season

Analyses carried out within individual years of the samples taken showed that in 2019 there were no significant differences between seasons in terms of volatile compounds aliphatics (*p* = 0.420), aromatics (*p* = 0.062), monoterpenes (*p* = 0.097), monoterpenoids (*p* = 0.368), and sesquiterpenoids (*p* = 0.717). However, a statistically significant effect of the difference between seasons in terms of undefined relationships was confirmed, F = 7.87; df = 2; *p* = 0.020, and sesquiterpenes, F = 11.31; df = 2; *p* = 0.003.

**Figure 4:**
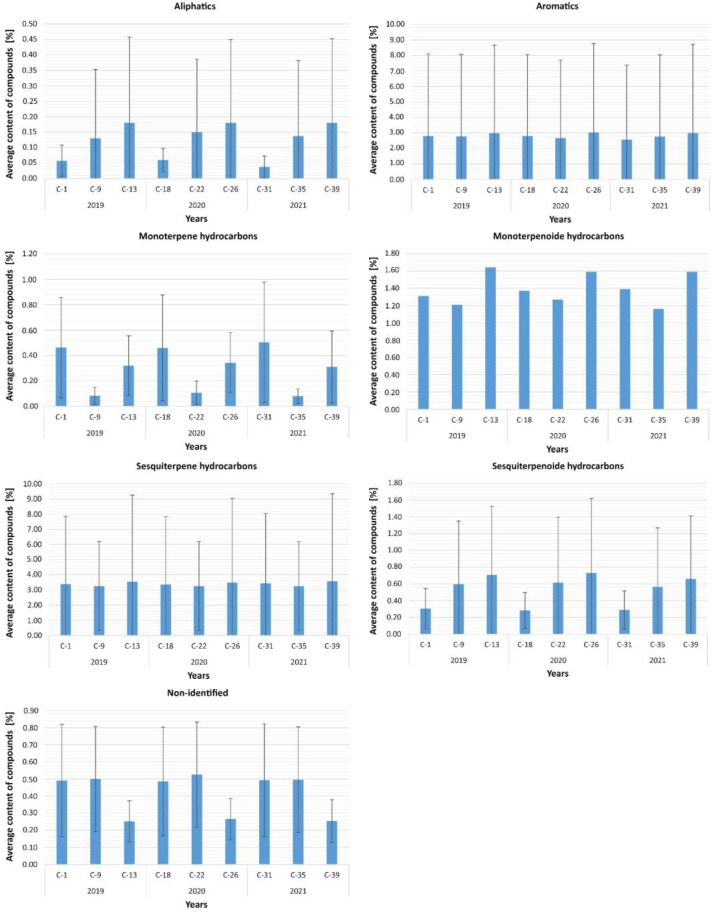
Average content of volatile organic compounds depending on the season.

As it turned out, the level of volatile compounds not identified was significantly higher in condition C-1 (*p* = 0.040) and was biased higher in condition C-9 (*p* = 0.058) compared to condition C-13. Furthermore, condition C-13 for sesquiterpene compounds showed higher levels of volatile compounds compared to C-9 (*p* = 0.003). No statistically significant differences were found in the other conditions in 2019 (Figure 4).

Similarly to 2019, samples collected in 2020 showed statistically significant differences only for volatile sesquiterpene compounds, F = 9.64; df = 2; *p* = 0.008, and non-identified compounds, F = 10.87; df = 2; *p* = 0.004. No significant differences were found between seasons in 2020 for the remaining compounds: aliphatics (*p* = 0.779), aromatics (*p* = 0.449), monoterpenes (*p* = 0.368), monoterpenoids (*p* = 0.368), and sesquiterpenoids (*p* = 0.717). Analysis of pairwise comparisons between seasons in terms of sesquiterpenes and differences not identified showed that the level of undefined compound in sample C-22 was significantly higher than in sample C-26 (*p* = 0.004). In contrast, the intensity of sesquiterpene compounds in sample C-22 was significantly lower than in sample C-26 (*p* = 0.006). No significant differences were found in the other conditions.

In 2021, the comparisons showed statistically significant differences in volatile sesquiterpenes, F = 7.86; df = 2; *p* = 0.020, and non-identified compounds, F = 9.16; df = 2; *p* = 0.010. The results of the analyses confirmed that the compounds not identified had higher levels in C-35 compared to C-39 (*p* = 0.012), while the sesquiterpene compounds had significantly lower severity in C-35 compared to C-39 (*p* = 0.027). No differences were found between C-31 and the other seasons. There were also no significant differences between seasons in 2021 for the remaining compounds: aliphatics (*p* = 0.424), aromatics (*p* = 0.486), monoterpenes (*p* = 0.529), monoterpenoids (*p* = 0.368), and sesquiterpenoids (*p* = 0.717).

Furthermore, an analysis of differences was performed using the Kruskal–Wallis test for the severity of individual volatile compounds in each section from C-9 to C-39 separately to confirm the possibility that individual compounds were more prevalent. As it turned out, statistically significant differences were found for C-9 in 2019, H = 13.45; df = 6; *p* = 0.036, also C-22 in 2020, H = 14.09; df = 6; *p* = 0.029, and C-35 in 2021, H = 13.45; df = 6; *p* = 0.036. Analysis of pairwise comparisons showed that the severity of sesquiterpene compounds was always significantly higher compared to aliphatic compounds, and these differences were significant at the level of *p* < 0.05 level. No significant differences were found for the other volatile compounds.

#### 2.6.2. Observation of Changes Depending on Storage

**Figure 5:**
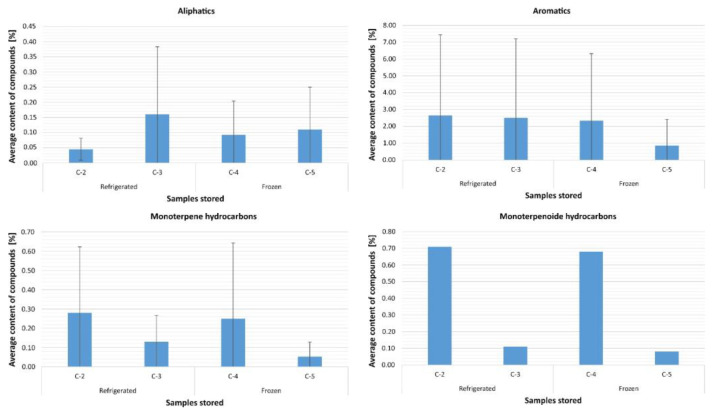

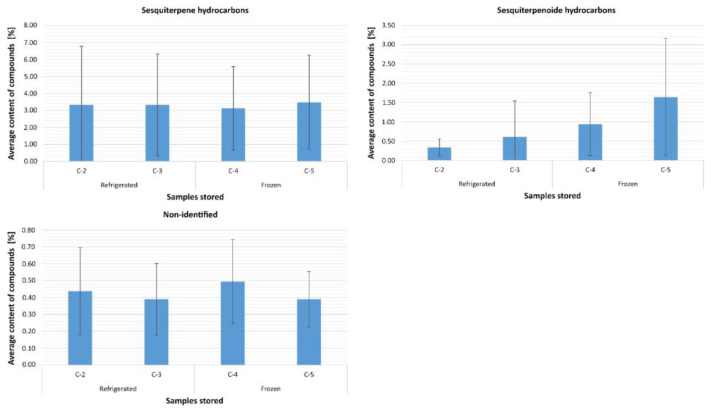
Average content of volatile compounds depending on the storage method.

Figure 5 shows the average content of volatile compounds depending on the storage method. Analysis of differences between all individual storage methods, taking into account the duration of use of a given method, showed statistically significant differences only between sesquiterpene compounds, F = 20.58; df = 3; *p* < 0.001.

Pairwise comparisons showed that the severity of the relationship was higher in condition C-5 compared to condition C-2 (*p* = 0.005) and C-3 (*p* = 0.030), and also lower in condition C-4 compared to condition C-2 (*p* = 0.003) and C-3 (*p* = 0.017). There were no significant differences between conditions C-2 and C-3, as well as conditions C-4 and C-5. Furthermore, it was confirmed that there were no differences in the storage of volatile compounds such as aliphatics (*p* = 0.801), aromatics (*p* = 0.266), monoterpenes (*p* = 0.072), monoterpenoids (*p* = 0.392), and sesquiterpenoids (*p* = 0.122). Additional analysis of the differences between the volatile compounds themselves under particular conditions showed significant differences in the case of condition C-2, H = 14, 62; df = 6; *p* = 0.023, and for condition C-4, H = 19.08; df = 6; *p* = 0.004. As it turned out, volatile sesquiterpene compounds under both conditions showed a higher intensity compared to aliphatic compounds, and these were the only significant differences between all volatile compounds at *p* < 0.05.

#### 2.6.3. Observation of Changes Depending on the Type of Substrate

**Figure 6:**
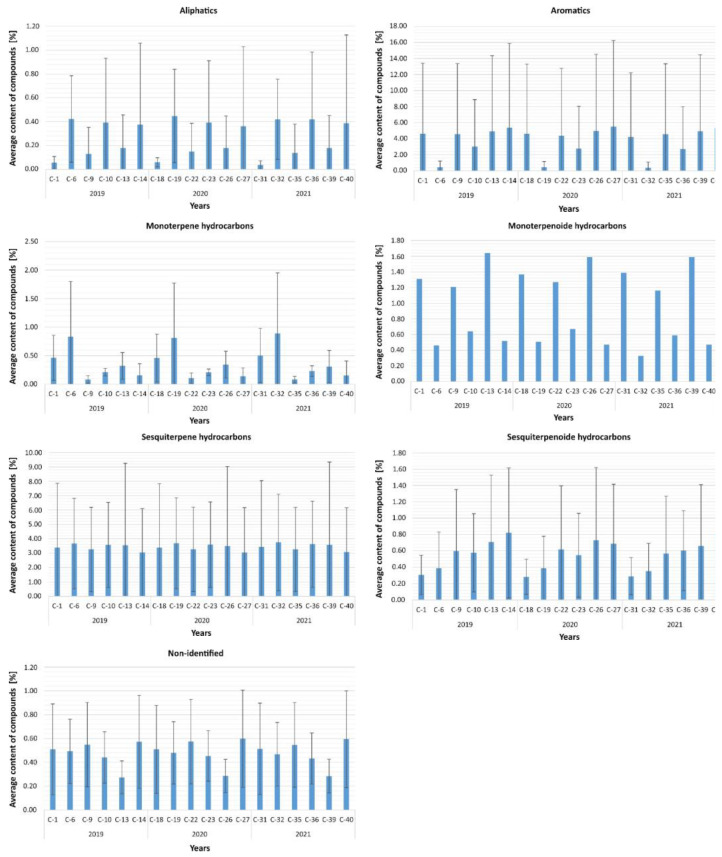
Varying the level of volatile compounds depending on the type of substrate.

Analyses conducted within each sampling year showed that in 2019, there were no significant differences between the substrates for volatile compounds: aliphatics (*p* = 0.178), aromatics (*p* = 0.142), monoterpenes (*p* = 0.119), monoterpenoids (*p* = 0.416), non-identified compounds (*p* = 0.126), and sesquiterpenoids (*p* = 0.885). However, sesquiterpenes were confirmed, F = 29.62; df = 5; *p* < 0.001. As it turned out, the measurement in period C-6 was statistically significantly higher compared to C-1 (*p* = 0.004), C-13 (*p* < 0.001), and C-14 (*p* = 0.008); in addition, the measurement of C-13 was higher compared to C-10 (*p* = 0.020). No statistically significant differences were found in the other conditions (Figure 6).

In 2020, they showed statistically significant differences in terms of volatile sesquiterpene compounds, F = 29.03; df = 5; *p* < 0.001, and non-identified compounds, F = 11.46; df = 5; *p* = 0.043. There were no significant differences between seasons in 2020 for the remaining compounds: aliphatics (*p* = 0.207), aromatics (*p* = 0.350), monoterpenes (*p* = 0.221), monoterpenoids (*p* = 0.416), and sesquiterpenoids (*p* = 0.982). Analysis of pairwise comparisons between different substrates in terms of differences between sesquiterpenes and non-identified compounds showed that the level of undefined compound in the C-22 segment was significantly higher than in the C-26 segment (*p* = 0.018). However, the intensity of sesquiterpene compounds in the C-19 segment was significantly higher than in C-18 (*p* = 0.013), C-26 (*p* < 0.001), and C-27 (*p* = 0.002), and, in addition, the compound level in C-26 was lower than in C-23 (*p* = 0.035). No significant differences were found in the remaining conditions. In 2021, the comparisons showed statistically significant differences only for volatile sesquiterpene compounds, F = 26.27; df = 5; *p* < 0.001. The results of the analyses confirmed that the sesquiterpene compounds had a significantly lower intensity in the C-31 (*p* = 0.005) and C-39 (*p* < 0.001) segment compared to C-32; moreover, in the C-39 condition, a lower level of sesquiterpene was found compared to C-36 (*p* = 0.026). There were also no significant differences in 2021 for the remaining compounds: aliphatics (*p* = 0.609), aromatics (*p* = 0.152), monoterpenes (*p* = 0.371), monoterpenoids (*p* = 0.416), not identified (*p* = 0.118), and sesquiterpenoid (*p* = 0.885).

Additionally, the analysis of differences was performed using the Kruskal–Wallis test in terms of the intensity of individual volatile compounds in each section from C-1 to C-40 for the conditions including Krzyżowa Pass and Krzyżowa Mount to confirm the possibility of a more frequent occurrence of individual compounds. The analysis showed significant statistical differences in 2019 for the condition C-6, H = 13.23; df = 6; *p* = 0.040 and for the condition C-9, H = 13.45; df = 6; *p* = 0.036. It turned out that in condition C-6, sesquiterpenes were biased more often than not identified (*p* = 0.053), while in condition C-9, sesquiterpenes were significantly more frequent compared to aliphatics (*p* = 0.048). In 2020, significant differences were confirmed in conditions C-19, H = 13.19; df = 6, *p* = 0.040 and also C-22, H = 14.09; df = 6; *p* = 0.029. Sesquiterpenes were significantly more frequent in the C-19 condition compared to those not identified (*p* = 0.048) and were more frequent in the C-22 condition compared to aliphatics (*p* = 0.033).

As in previous years, in 2021, significant differences were also found in two conditions: C-32, H = 13.82; df = 6; *p* = 0.032 and C-35, H = 13.45; df = 6; *p* = 0.036. As it turned out, a higher intensity of sesquiterpene compounds in the C-32 condition compared to not identified compounds (*p* = 0.028) was confirmed, as well as a higher level of sesquiterpenes in the C-35 condition compared to aliphatics.

## 3. Materials and Methods

### 3.1. Plant Material

The plant material studied included 43 *Calypogeia azurea* samples cultured and obtained from habitats in different regions of Poland (Table 1). Natural liverwort samples were collected in the years 2019–2021 in three seasons: spring, summer, and autumn. Research materials were collected at three locations in the Karkonosze and Beskid Sądecki regions near Karpacz, Szklarska Poręba, and Krynica Zdrój at 700–1200 m A.S.L. All samples, except those from Krzyżowa Góra, were taken from the slope near the frequented routes. Plants collected on Góra Krzyżowa grew on fallen, rotten trees and rotting trees under forest litter. Five samples weighing approximately 15 g each were taken from each natural site. Only green plants that did not show signs of drying out and were not affected by visible diseases were eligible for collection and further research. In natural habitats, liverwort samples are initially identified on the basis of their morphological structure. In the laboratory, samples of *C. azurea* identified as species based on six DNA barcodes. Before analysis, the samples were cleaned from different plant material and soil. Research was conducted on fresh material. For selected samples from Beskid Sądecki, the impact of storage on VOC was analysed. For this purpose, the collected research material was stored for 1 month and 3 months in a refrigerator at a temperature of 5 °C and in a freezer at a temperature of −30 °C.

### 3.2. HS-SPME Extraction

Volatile compounds from *Calypogeia azurea* were extracted by the headspace solid phase microextraction technique. Fused silica fibres coated with divinylbenzene/carboxen/polydimethylsiloxane (DVB/CAR/PDMS) were used. A 2-cm long fibre covered with a 50/30 µm thick film was used. Before analysis, the fibres were conditioned for 1 h at 270 °C, according to the supplier’s instructions. Then, 5 mg of clean and dried plant material was placed in a 1.7 mL vial hermetically closed with a Teflon/silicone septum and heated at 50 °C. The extraction of the compounds was followed at 50 °C for 60 min. Fibre analyte desorption was carried out in the injection port of the gas chromatograph at 250 °C for 10 min. Sorption and desorption operations were performed using the TriPlus RSH autosampler (Thermo Scientific, Waltham, MA, USA).

### 3.3. GC-MS Analysis

The analysis of volatile compounds was performed using a previously described GC-MS method [30,31]. GC-MS analyses using a silphenylene phase were performed on a Trace 1310 (Thermo Scientific, Waltham, MA, USA) equipped with a Quadrex 007-5MS column (30 m, 0.25 mm, 0.25 μm). The ISQ QD mass detector (Thermo Scientific, Waltham, MA, USA) was operated at 70 eV in the EI mode in the m/z range of 30 to 550. This was used as the carrier gas at a flow rate of 1.0 mL/min. The oven temperature was programmed from 60 to 230 °C at 4 °C/min and then isothermal at 230 °C for 40 min. The injector temperature and transfer line were 250 °C. Injection samples were in splitless mode with a dedicated liner for the SPME technique. The identification of components was confirmed by comparing the mass spectral fragmentation patterns with those stored in the MS database (NIST 2011, NIST Chemistry WebBook, Adams 4 Library, MassFinder 4, and Pherobase) and those reported in the literature [32,33]. Furthermore, retention indices on nonpolar columns, determined relative to a homologous series of n-alkanes (C7–C40), were compared with the data of the published indices. Quantitative data of the components were obtained by integrating the TIC chromatogram and calculating the relative percentage of the peak areas. Each sample of *Calypogeia azurea* was analysed three times.

### 3.4. Statistical Analysis

The methods used were Friedman’s ANOVA and Kurskal–Wallis rank tests because of the high intensity of variance. By transforming the results to a unified scale, rank analyses allowed more accurate comparisons to be made without accounting for measurement error for the means. A threshold of α = 0.05 was used as the significance level. The determination of the statistical analysis conditions is shown in Table 1, Table 2, Table 3, Table 4, Table 5, Table 6, Table 7, Table 8, Table 9 and Table 10. The paper discusses the results of the statistical analysis performed on the example of samples for the location of the Krzyżowa Pass, because similar relationships were observed in other locations.

## 4. Conclusions

This study examined whether and to what extent *Calypogeia* liverworts are susceptible to environmental stress. A total of 43 samples collected at different times of the year and samples grown in vitro were analysed. In these samples, 79 volatile organic compounds were detected, 47 of which were identified. Knowing that the content of specialized metabolites varies with the seasons, *Calypogeia azurea* has been characterized in three different seasons (spring, summer, and autumn). Since winter is considered the dormant period for plants, including liverworts, the winter season has not been included in the present study.

Based on the VOC composition tests performed, it was found that *Calypogeia azurea* is a liverwort species particularly susceptible to stress and plant defence reactions. The biosynthesis of specialized metabolites is induced by environmental conditions. Studies have shown that abiotic factors such as sample storage and freezing and the type of medium on which *Calypogeia azurea* grows influence the production of specialized metabolites. Sunlight and humidity increase or decrease the percentage of volatile organic compounds present in plant cells. On the other hand, *C. azurea* obtained from in vitro culture is similar in terms of VOC composition to plant material obtained in summer from natural sites.

Due to their low morphology, liverworts are difficult to classify and identify. As they are rich in volatile organic compounds, they can be used to evaluate their chemosystematics. The conducted research shows that storing the sample in a refrigerator or a freezer may cause changes in the content of volatile organic compounds, which is why it is so important to test liverworts on fresh samples.

## Figures and Tables

**Table 1:** The *Calypogeia azurea* sampling data used for studies.

Sample Code	Collection Place	Geographical Coordinates	Type of Sample	Date Month Year
C-1	SE, Poland, Beskid Sądecki Mts, Krzyżowa Pass	49°25′24.7″ N, 20°56′01.4″ E	fresh	May 2019
C-2	SE Poland, Beskid Sądecki Mts, Krzyżowa Pass	49°25′24.7″ N, 20°56′01.4″ E	fridge 1 month	May 2019
C-3	SE Poland, Beskid Sądecki Mts, Krzyżowa Pass	49°25′24.7″ N, 20°56′01.4″ E	fridge 3 months	May 2019
C-4	SE Poland, Beskid Sądecki Mts, Krzyżowa Pass	49°25′24.7″ N, 20°56′01.4″ E	freezer 1 month	May 2019
C-5	SE Poland, Beskid Sądecki Mts, Krzyżowa Pass	49°25′24.7″ N, 20°56′01.4″ E	freezer 3 months	May 2019
C-6	SE, Poland, Beskid Sądecki Mts, Krzyżowa Mount	49°25′09.1″ N, 20°56′24.8″ E	fresh	May 2019
C-7	SW Poland, Karkonosze Mts, Sowia Valley	50°45′19.1″ N, 15°46′32.1″ E	fresh	May 2019
C-8	SW Poland, Karkonosze Mts, Stara Road	50°47′52.9″ N, 15°31′41.8″ E	fresh	May 2019
C-9	SE Poland, Beskid Sądecki Mts, Krzyżowa Pass	49°25′24.7″ N, 20°56′01.4″ E	fresh	August 2019
C-10	SE, Poland, Beskid Sądecki Mts, Krzyżowa Mount	49°25′09.1″ N, 20°56′24.8″ E	fresh	August 2019
C-11	SW Poland, Karkonosze Mts, Sowia Valley	50°45′19.1″ N, 15°46′32.1″ E	fresh	August 2019
C-12	SW Poland, Karkonosze Mts, Stara Road	50°47′52.9″ N, 15°31′41.8″ E	fresh	August 2019
C-13	SE Poland, Beskid Sądecki Mts, Krzyżowa Pass	49°25′24.7″ N, 20°56′01.4″ E	fresh	November 2019
C-14	SE, Poland, Beskid Sądecki Mts, Krzyżowa Mount	49°25′09.1″ N, 20°56′24.8″ E	fresh	November 2019
C-15	SW Poland, Karkonosze Mts, Sowia Valley	50°45′19.1″ N, 15°46′32.1″ E	fresh	November 2019
C-16	SW Poland, Karkonosze Mts, Stara Road	50°47′52.9″ N, 15°31′41.8″ E	fresh	November 2019
C-17	SE, Poland, Beskid Sądecki Mts, Krzyżowa Pass	49°25′24.7″ N, 20°56′01.4″ E	in vitro	November 2019
C-18	SE, Poland, Beskid Sądecki Mts, Krzyżowa Pass	49°25′24.7″ N, 20°56′01.4″ E	fresh	May 2020
C-19	SE, Poland, Beskid Sądecki Mts, Krzyżowa Mount	49°25′24.7″ N, 20°56′01.4″ E	fresh	May 2020
C-20	SW Poland, Karkonosze Mts, Sowia Valley	50°45′19.1″ N, 15°46′32.1″ E	fresh	May 2020
C-21	SW Poland, Karkonosze Mts, Stara Road	50°47′52.9″ N, 15°31′41.8″ E	fresh	May 2020
C-22	SE Poland, Beskid Sądecki Mts, Krzyżowa Pass	49°25′24.7″ N, 20°56′01.4″ E	fresh	August 2020
C-23	SE, Poland, Beskid Sądecki Mts, Krzyżowa Mount	49°25′09.1″ N, 20°56′24.8″ E	fresh	August 2020
C-24	SW Poland, Karkonosze Mts, Sowia Valley	50°45′19.1″ N, 15°46′32.1″ E	fresh	August 2020
C-25	SW Poland, Karkonosze Mts, Stara Road	50°47′52.9″ N, 15°31′41.8″ E	fresh	August 2020
C-26	SE, Poland, Beskid Sądecki Mts, Krzyżowa Pass	49°25′24.7″ N, 20°56′01.4″ E	fresh	November 2020
C-27	SE, Poland, Beskid Sądecki Mts, Krzyżowa Mount	49°25′09.1″ N, 20°56′24.8″ E	fresh	November 2020
C-28	SW Poland, Karkonosze Mts, Sowia Valley	50°45′19.1″ N, 15°46′32.1″ E	fresh	November 2020
C-29	SW Poland, Karkonosze Mts, Stara Road	50°47′52.9″ N, 15°31′41.8″ E	fresh	November 2020
C-30	SE, Poland, Beskid Sądecki Mts, Krzyżowa Pass	49°25′24.7″ N, 20°56′01.4″ E	in vitro	November 2020
C-31	SE, Poland, Beskid Sądecki Mts, Krzyżowa Pass	49°25′24.7″ N, 20°56′01.4″ E	fresh	May 2021
C-32	SE, Poland, Beskid Sądecki Mts, Krzyżowa Mount	49°25′24.7″ N, 20°56′01.4″ E	fresh	May 2021
C-33	SW Poland, Karkonosze Mts, Sowia Valley	50°45′19.1″ N, 15°46′32.1″ E	fresh	May 2021
C-34	SW Poland, Karkonosze Mts, Stara Road	50°47′52.9″ N, 15°31′41.8″ E	fresh	May 2021
C-35	SE Poland, Beskid Sądecki Mts, Krzyżowa Pass	49°25′24.7″ N, 20°56′01.4″ E	fresh	August 2021
C-36	SE, Poland, Beskid Sądecki Mts, Krzyżowa Mount	49°25′09.1″ N, 20°56′24.8″ E	fresh	August 2021
C-37	SW Poland, Karkonosze Mts, Sowia Valley	50°45′19.1″ N, 15°46′32.1″ E	fresh	August 2021
C-38	SW Poland, Karkonosze Mts, Stara Road	50°47′52.9″ N, 15°31′41.8″ E	fresh	August 2021
C-39	SE Poland, Beskid Sądecki Mts, Krzyżowa Pass	49°25′24.7″ N, 20°56′01.4″ E	fresh	November 2021
C-40	SE, Poland, Beskid Sądecki Mts, Krzyżowa Mount	49°25′09.1″ N, 20°56′24.8″ E	fresh	November 2021
C-41	SW Poland, Karkonosze Mts, Sowia Valley	50°45′19.1″ N, 15°46′32.1″ E	fresh	November 2021
C-42	SW Poland, Karkonosze Mts, Stara Road	50°47′52.9″ N, 15°31′41.8″ E	fresh	November 2021
C-43	SE, Poland, Beskid Sądecki Mts, Krzyżowa Pass	49°25′24.7″ N, 20°56′01.4″ E	in vitro	November 2021

**Table 2:** Volatile compounds detected in the samples C-1–C-5.

No.	Compounds	RI ^a^	Code ^b^				
			C-1	C-2	C-3	C-4	C-5
1	2-methyl-1-propanol	<700	-	-	-	-	-
2	pentanal	<700	0.13 (0.02)	0.10 (0.02)	0.05 (0.01)	0.07 (0.03)	0.03 (0.01)
3	cyclopentanol	748	-	-	-	-	-
4	3-methyl-1-butanol	768	0.04 (0.02)	0.02 (0.02)	0.50 (0.08)	0.26 (0.08)	0.32 (0.08)
5	hexanal	834	0.05 (0.01)	0.03 (0.02)	0.02 (0.01)	0.02 (0.01)	0.01 (*)
6	(*Z*)-3-hexen-1-ol	886	-	-	-	0.04 (0.01)	0.11 (0.04)
7	1-hexanol	896	-	-	-	0.06 (0.02)	0.11 (0.03)
8	106[M+](58) 91(100) 77(13)	902	0.79 (0.06)	0.59 (0.07)	0.11 (0.04)	0.35 (0.11)	0.02 (0.01)
9	tricyclene	939	-	-	-	-	-
10	α-pinene	952	0.06 (0.01)	0.04 (0.02)	0.02 (0.01)	0.03 (0.01)	0.01 (*)
11	camphene	971	-	-	-	-	-
12	β-pinene	1004	0.66 (0.01)	0.18 (0.03)	0.11 (0.04)	0.07 (0.02)	0.02 (0.01)
13	3-octanol	1018	-	0.13 (0.02)	-	0.15 (0.11)	-
14	limonene	1048	0.67 (0.01)	0.62 (0.02)	0.26 (0.05)	0.65 (0.21)	0.13 (0.04)
15	benzeneacetaldehyde	1093	0.12 (0.02)	0.33 (0.02)	0.09 (0.02)	0.48 (0.12)	0.01 (*)
16	1-octen-3-yl-acetate	1123	0.01 (*)	0.03 (0.02)	0.07 (0.02)	0.02 (0.01)	0.08 (0.02)
17	benzeneethanol	1156	0.01 (*)	0.21 (0.02)	0.02 (0.01)	0.55 (0.11)	0.04 (0.01)
18	160[M+](49) 145(100) 117(34)	1224	4.49 (0.05)	3.49 (0.05)	2.63 (0.11)	3.36 (1.14)	0.51 (0.11)
19	β-cyclocitral	1257	1.31 (0.03)	0.71 (0.04)	0.11 (0.03)	0.68 (0.12)	0.08 (0.02)
20	1,2-dihydro-6-methylnaphthalene	1295	0.14 (0.02)	0.08 (0.03)	0.05 (0.01)	0.04 (0.02)	0.03 (0.01)
21	207[M]+(18) 121(100) 93(71)	1332	0.03 (0.02)	0.05 (0.02)	0.07 (0.04)	0.06 (0.01)	0.13 (0.08)
22	δ-elemene	1344	0.76 (0.02)	1.34 (0.04)	1.52 (0.21)	1.31 (0.21)	2.21 (1.11)
23	methylnaphthalene	1359	0.15 (0.02)	0.17 (0.03)	0.27 (0.08)	0.15 (0.11)	0.19 (0.08)
24	202[M+](18) 81(100) 96(83)	1364	0.09 (0.02)	0.15 (0.03)	0.16 (0.05)	0.21 (0.08)	0.46 (0.11)
25	anastreptene	1379	6.72 (0.03)	10.72 (0.10)	14.40 (1.01)	23.93 (1.12)	26.43 (1.34)
26	204[M+](25) 105(100) 161(83)	1382	0.52 (0.02)	0.24 (0.02)	0.08 (0.02)	0.11 (0.04)	0.04 (0.01)
27	202[M+](5) 159(100) 91(95)	1387	0.10 (0.10)	0.08 (0.03)	0.05 (0.01)	0.07 (0.02)	0.03 (0.01)
28	202[M+](5) 143(100) 128(92)	1392	1.73 (0.03)	1.63 (0.05)	1.35 (0.03)	0.75 (0.12)	0.07 (0.02)
29	β-elemene	1405	0.18 (0.02)	0.38 (0.04)	0.38 (0.01)	0.59 (0.08)	0.93 (0.21)
30	204[M]+(52) 161(100) 107(89)	1407	-	-	-	-	-
31	202[M]+(2) 143(100) 128(93)	1418	0.28 (0.03)	0.24 (0.02)	0.19 (0.01)	0.06 (0.02)	0.04 (0.01)
32	α-gurjunene	1423	0.20 (0.01)	0.21 (0.02)	0.25 (0.02)	0.26 (0.08)	0.28 (0.09)
33	202[M]+(2) 145(100) 160(35)	1426	0.22 (0.02)	0.18 (0.02)	0.15 (0.01)	0.20 (0.06)	0.18 (0.07)
34	β-gurjunene	1431	0.20 (0.02)	0.26 (0.03)	0.15 (0.03)	0.52 (0.12)	0.11 (0.05)
35	204[M]+(12) 159(100) 105(95)	1436	0.13 (0.02)	0.11 (0.02)	0.12 (0.02)	0.21 (0.08)	0.31 (0.11)
36	(−)-aristolene	1439	0.29 (0.02)	0.23 (0.03)	0.17 (0.02)	0.26 (0.08)	0.11 (0.08)
37	γ-gurjunene	1444	0.04 (0.01)	0.03 (0.02)	0.04 (0.01)	0.03 (0.01)	0.02 (0.01)
38	γ-maaliene	1449	0.39 (0.02)	0.44 (0.02)	0.66 (0.03)	0.83 (0.14)	1.19 (0.21)
39	α-maaliene	1455	0.33 (0.02)	0.59 (0.04)	0.56 (0.03)	0.79 (0.18)	0.71 (0.11)
40	alloaromadendrene	1459	3.63 (0.04)	5.50 (0.20)	5.87 (0.06)	8.38 (2.32)	10.17 (2.46)
41	selina-5,11-diene	1470	0.56 (0.04)	0.90 (0.06)	0.64 (0.02)	1.73 (0.08)	1.63 (0.32)
42	202[M]+(20) 105(100) 159(68)	1475	0.64 (0.06)	0.71 (0.04)	0.85 (0.04)	0.99 (0.12)	1.52 (0.28)
43	202[M+](30) 159(100) 131(75)	1479	0.51 (0.01)	0.62 (0.02)	0.26 (0.03)	1.12 (0.06)	0.73 (0.11)
44	dehydroaromadendrene	1482	0.47 (0.06)	0.66 (0.03)	0.72 (0.06)	1.01 (0.04)	1.21 (0.22)
45	204[M+](1) 142(100) 141(78)	1495	0.11 (0.02)	0.09 (0.02)	0.05 (0.02)	0.29 (0.08)	0.31 (0.10)
46	β-guaiene	1505	0.04 (0.01)	0.07 (0.02)	0.13 (0.04)	0.11 (0.02)	0.08 (0.04)
47	germacrene D	1510	2.78 (0.14)	3.22 (0.05)	2.19 (0.11)	5.15 (1.21)	4.95 (1.11)
48	ledene	1516	0.03 (0.01)	0.06 (0.02)	0.04 (0.01)	0.13 (0.06)	0.09 (0.03)
49	bicyclogermacrene	1521	4.08 (0.31)	7.01 (0.12)	10.43 (0.31)	13.00 (2.42)	15.39 (5.43)
50	220[M+](5) 148(100) 133(95)	1525	0.12 (0.02)	0.36 (0.02)	0.26 (0.06)	0.72 (0.12)	0.43 (0.14)
51	cuparene	1538	0.68 (0.08)	1.06 (0.03)	1.58 (0.08)	1.85 (0.24)	3.82 (1.12)
52	204[M+](22) 93(100) 105(68)	1543	0.08 (0.03)	0.05 (0.03)	0.04 (0.01)	0.09 (0.02)	0.08 (0.02)
53	202[M+](12) 157(100) 142(62)	1554	0.36 (0.04)	0.45 (0.03)	0.31 (0.03)	1.52 (0.24)	1.20 (0.24)
54	204[M+](18) 155(100) 119(82)	1558	0.06 (0.02)	0.04 (0.02)	0.07 (0.02)	0.02 (0.01)	0.03 (0.01)
55	200[M]+ (72) 143(100) 129(93)	1571	0.22 (0.04)	0.45 (0.02)	0.42 (0.08)	0.64 (0.16)	0.85 (0.12)
56	200[M+](73) 143(100) 157(97)	1579	0.05 (0.02)	0.12 (0.02)	0.33 (0.04)	0.31 (0.10)	0.23 (0.10)
57	204[M]+ (6) 69(100) 41(85)	1586	0.22 (0.04)	0.05 (0.02)	0.05 (0.01)	0.08 (0.02)	0.06 (0.02)
58	cadina-3,9-diene	1591	0.21 (0.03)	0.19 (0.02)	0.12 (0.05)	0.27 (0.08)	0.21 (0.09)
59	maaliol	1609	-	-	-	0.25 (0.08)	1.52 (0.22)
60	1,4-dimethylazulene	1616	46.10 (1.40)	36.35 (0.91)	29.16 (1.22)	4.00 (0.32)	0.42 (0.10)
61	(+)-spathulenol	1619	-	0.2 (0.03)	-	0.21 (0.10)	-
62	(−)-globulol	1631	0.50 (0.07)	0.15 (0.03)	0.06 (0.01)	0.13 (0.06)	0.21 (0.09)
63	220[M+](5) 132(100) 43(96)	1637	0.47 (0.04)	0.47 (0.06)	0.93 (0.08)	1.21 (0.28)	1.28 (0.21)
64	202[M+](3) 166(100) 165(98)	1645	-	-	-	-	-
65	202[M+](2)129(100) 172(72)	1655	1.53 (0.22)	1.54 (0.06)	1.54 (0.13)	0.80 (0.11)	1.43 (0.18)
66	200[M+](2) 145(100) 158(55)	1664	-	-	-	-	-
67	isospathulenol	1667	0.34 (0.04)	0.32 (0.03)	0.21 (0.07)	1.17 (0.21)	1.85 (0.15)
68	218[M+](2) 71(100) 57(93)	1674	0.08 (0.02)	0.03 (0.01)	0.05 (0.02)	0.26 (0.08)	0.06 (0.02)
69	220[M+](5) 129(100) 144(92)	1678	0.10 (0.03)	0.08 (0.02)	0.05 (0.01)	0.07 (0.02)	0.05 (0.01)
70	220[M+](31) 159(100) 105(81)	1684	0.46 (0.04)	0.32 (0.02)	0.66 (0.08)	0.17 (0.07)	0.52 (0.10)
71	202[M+](92) 143(100) 128(78)	1689	0.13 (0.03)	0.21 (0.03)	0.15 (0.05)	0.08 (0.02)	0.05 (0.01)
72	220[M+](11) 109(100) 121(51)	1695	0.06 (0.02)	0.07 (0.02)	0.04 (0.01)	0.14 (0.08)	0.11 (0.08)
73	220[M+](13) 159(100) 145(89)	1710	0.16 (0.04)	0.12 (0.03)	0.12 (0.07)	0.18 (0.04)	0.24 (0.08)
74	4-(cyclopent-1-enyl)benzoic acid methyl ester	1722	13.61 (0.58)	12.43 (0.15)	12.08 (0.58)	10.45 (2.22)	4.04 (1.12)
75	216[M+](10) 202(100) 159(96)	1744	0.08 (0.02)	0.15 (0.03)	0.21 (0.08)	0.29 (0.08)	0.33 (0.12)
76	216[M+](3) 202(100) 143(81)	1784	0.05 (0.03)	0.07 (0.02)	0.03 (0.01)	-	-
77	germacra-4(15),5,10(14)-trien-1-alpha-ol	1801	0.42 (0.03)	0.67 (0.06)	0.82 (0.11)	1.26 (0.26)	2.89 (1.11)
78	1,4-dimethyl-7-(1-methylethyl)-azulene	1823	0.05 (0.03)	0.05 (0.02)	0.02 (0.01)	0.25 (0.10)	0.27 (0.09)
79	methyl-2-methylazulene-1-carboxylate	2025	0.08 (0.02)	0.54 (0.04)	1.56 (0.22)	1.52 (0.34)	2.87 (1.12)
	Total		99.91 (4.22)	98.99 (3.23)	96.66 (5.89)	97.02 (16.53)	96.08 (20.87)
	% Identified		86.04 (3.19)	86.23 (2.41)	85.33 (4.67)	82.66 (13.14)	84.78 (18.38)
	Including:						
	Aliphatics		0.23 (0.05)	0.31 (0.09)	0.64 (0.12)	0.62 (0.27)	0.66 (0.18)
	Aromatics		14.03 (0.63)	13.22 (0.24)	12.51 (0.68)	11.67 (2.55)	4.31 (1.22)
	Monoterpene hydrocarbons		1.39 (0.03)	0.84 (0.06)	0.39 (0.09)	0.75 (0.24)	0.16 (0.05)
	Monoterpenoide hydrocarbons		1.31 (0.03)	0.71 (0.04)	0.11 (0.03)	0.68 (0.12)	0.08 (0.02)
	Sesquiterpene hydrocarbons		68.16 (2.32)	69.94 (1.86)	69.85 (3.45)	65.66 (9.17)	73.12 (15.33)
	Sesquiterpenoide hydrocarbons		0.92 (0.14)	1.21 (0.12)	1.83 (0.30)	3.28 (0.79)	6.45 (1.58)

-, * less than 0.01%. ^a^ Retention index on Quadex 007-5MS column. ^b^ For abbreviations of samples see Table 1. ( ) standard deviation.

**Table 3:** Volatile compounds detected in the samples C-6–C-10.

No.	Compounds	RI ^a^	Code ^b^				
			C-6	C-7	C-8	C-9	C-10
1	2-methyl-1-propanol	<700	0.15 (0.08)	-	-	-	0.08 (0.02)
2	pentanal	<700	0.05 (0.01)	0.14 (0.02)	0.06 (0.02)	0.01 (*)	0.03 (0.01)
3	cyclopentanol	748	0.05 (0.02)	-	-	-	0.06 (0.01)
4	3-methyl-1-butanol	768	0.45 (0.11)	0.03 (0.01)	0.02 (0.01)	0.03 (0.01)	0.21 (0.09)
5	hexanal	834	0.27 (0.08)	0.02 (0.01)	0.01 (*)	0.01 (*)	0.12 (0.04)
6	(*Z*)-3-hexen-1-ol	886	0.16 (0.06)	-	-	-	0.10 (0.02)
7	1-hexanol	896	0.08 (0.04)	-	-	0.10 (0.02)	0.12 (0.04)
8	106[M+](58) 91(100) 77(13)	902	1.22 (0.22)	0.65 (0.08)	0.55 (0.10)	0.41 (010)	0.60 (0.10)
9	tricyclene	939	0.09 (0.02)	-	-	-	0.05 (0.01)
10	α-pinene	952	0.37 (0.08)	0.05 (0.01)	0.08 (0.02)	0.04 (0.01)	0.18 (0.04)
11	camphene	971	0.70 (0.12)	-	-	-	0.48 (0.11)
12	β-pinene	1004	0.31 (0.10)	0.61 (0.07)	0.53 (0.12)	0.06 (0.01)	0.18 (0.06)
13	3-octanol	1018	0.15 (0.08)	-	-	0.03 (0.01)	0.23 (0.09)
14	limonene	1048	1.82 (0.21)	0.45 (0.06)	0.56 (0.10)	0.15 (0.06)	0.28 (0.09)
15	benzeneacetaldehyde	1093	0.07 (0.02)	0.21 (0.04)	0.15 (0.04)	0.07 (0.01)	0.05 (0.01)
16	1-octen-3-yl-acetate	1123	0.92 (0.16)	0.02 (0.01)	0.05 (0.04)	0.47 (0.10)	1.21 (0.09)
17	benzeneethanol	1156	-	0.01 (*)	0.01 (*)	0.01 (*)	-
18	160[M+](49) 145(100) 117(34)	1224	2.33 (0.88)	3.43 (0.12)	4.90 (1.11)	3.49 (0.88)	2.23 (0.21)
19	β-cyclocitral	1257	0.46 (0.10)	1.45 (0.10)	1.28 (0.24)	1.21 (0.10)	0.64 (0.10)
20	1,2-dihydro-6-methylnaphthalene	1295	-	0.10 (0.04)	0.04 (0.01)	0.10 (0.03)	-
21	207[M]+(18) 121(100) 93(71)	1332	0.06 (0.02)	0.09 (0.02)	0.10 (0.02)	0.14 (0.08)	0.07 (0.01)
22	δ-elemene	1344	1.17 (0.34)	0.66 (0.08)	0.42 (0.10)	2.32 (0.21)	1.21 (0.09)
23	methylnaphthalene	1359	0.01 (*)	0.08 (0.02)	0.08 (0.02)	0.09 (0.02)	0.03 (0.01)
24	202[M+](18) 81(100) 96(83)	1364	0.35 (0.10)	0.08 (0.02)	0.12 (0.04)	0.19 (0.08)	0.15 (0.04)
25	anastreptene	1379	30.89 (1.58)	6.92 (0.12)	6.82 (1.24)	11.95 (2.22)	21.06 (3.34)
26	204[M+](25) 105(100) 161(83)	1382	-	0.44 (0.08)	0.49 (0.12)	0.32 (0.10)	0.05 (0.01)
27	202[M+](5) 159(100) 91(95)	1387	-	0.16 (0.03)	0.05 (0.01)	0.05 (0.01)	-
28	202[M+](5) 143(100) 128(92)	1392	0.45 (0.11)	1.82 (0.11)	1.44 (0.34)	1.21 (0.08)	0.55 (0.12)
29	β-elemene	1405	0.47 (0.10)	0.20 (0.08)	0.43 (0.08)	0.50 (0.10)	0.41 (0.10)
30	204[M]+(52) 161(100) 107(89)	1407	0.01 (*)	-	-	-	0.04 (0.01)
31	202[M]+(2) 143(100) 128(93)	1418	0.17 (0.08)	0.38 (0.10)	0.20 (0.09)	0.21 (0.09)	0.09 (0.02)
32	α-gurjunene	1423	0.69 (0.10)	0.20 (0.08)	0.13 (0.06)	0.25 (0.08)	0.29 (0.09)
33	202[M]+(2) 145(100) 160(35)	1426	0.01 (0.09)	0.35 (0.04)	0.25 (0.08)	0.18 (0.06)	0.11 (0.04)
34	β-gurjunene	1431	0.46 (0.10)	0.15 (0.04)	0.13 (0.07)	0.05 (0.01)	0.07 (0.02)
35	204[M]+(12) 159(100) 105(95)	1436	0.24 (0.09)	0.12 (0.03)	0.12 (0.08)	0.03 (0.01)	0.25 (0.09)
36	(−)-aristolene	1439	0.37 (0.10)	0.31 (0.09)	0.21 (0.09)	0.34 (0.09)	0.15 (0.04)
37	γ-gurjunene	1444	0.14 (0.08)	0.04 (0.01)	0.05 (0.01)	0.05 (0.01)	0.09 (0.02)
38	γ-maaliene	1449	0.83 (0.12)	0.92 (0.08)	0.78 (0.10)	0.60 (0.10)	0.74 (0.10)
39	α-maaliene	1455	0.78 (0.10)	0.35 (0.06)	0.45 (0.09)	0.39 (0.09)	0.62 (0.09)
40	alloaromadendrene	1459	8.83 (1.22)	3.32 (0.12)	4.32 (0.14)	4.70 (1.10)	6.22 (1.22)
41	selina-5,11-diene	1470	1.07 (0.21)	0.92 (0.08)	0.92 (0.21)	0.81 (0.10)	0.24 (0.09)
42	202[M]+(20) 105(100) 159(68)	1475	1.61 (0.32)	0.19 (0.03)	0.16 (0.06)	0.14 (0.07)	0.82 (0.12)
43	202[M+](30) 159(100) 131(75)	1479	2.20 (0.43)	0.62 (0.12)	0.18 (0.07)	0.28 (0.09)	1.14 (0.21)
44	dehydroaromadendrene	1482	1.47 (0.32)	0.65 (0.10)	0.53 (0.10)	1.00 (0.22)	0.88 (0.09)
45	204[M+](1) 142(100) 141(78)	1495	0.16 (0.08)	0.16 (0.06)	0.08 (0.02)	0.10 (0.03)	0.30 (0.05)
46	β-guaiene	1505	0.08 (0.04)	0.03 (0.01)	0.04 (0.01)	0.14 (0.04)	0.20 (0.07)
47	germacrene D	1510	5.22 (1.05)	3.48 (0.12)	3.20 (0.22)	2.82 (0.22)	3.21 (0.32)
48	ledene	1516	0.13 (0.04)	-	-	0.03 (0.01)	0.05 (0.01)
49	bicyclogermacrene	1521	8.93 (1.73)	4.52 (1.11)	4.32(0.84)	9.63 (1.11)	15.59 (1.34)
50	220[M+](5) 148(100) 133(95)	1525	0.10 (0.04)	0.25 (0.07)	0.22 (0.09)	0.52 (0.10)	0.42 (0.09)
51	cuparene	1538	1.94 (0.32)	0.78 (0.10)	0.65 (0.10)	1.47 (0.21)	1.02 (0.12)
52	204[M+](22) 93(100) 105(68)	1543	0.01 (0.04)	0.08 (0.02)	0.08 (0.02)	0.05 (0.01)	0.08 (0.02)
53	202[M+](12) 157(100) 142(62)	1554	1.31 (0.22)	0.37 (0.08)	0.18 (0.08)	0.49 (0.10)	0.20 (0.09)
54	204[M+](18) 155(100) 119(82)	1558	0.09 (0.02)	0.09 (0.02)	0.02 (0.01)	0.03 (0.01)	0.05 (0.01)
55	200[M]+ (72) 143(100) 129(93)	1571	0.42 (0.12)	0.32 (0.09)	0.32 (0.09)	0.79 (0.12)	0.36 (0.11)
56	200[M+](73) 143(100) 157(97)	1579	0.22 (0.08)	0.08 (0.02)	0.06 (0.01)	0.23 (0.08)	0.15 (0.08)
57	204[M]+ (6) 69(100) 41(85)	1586	0.06 (0.02)	0.30 (0.09)	0.11 (0.02)	0.15 (0.08)	0.08 (0.02)
58	cadina-3,9-diene	1591	0.11 (0.04)	0.15 (0.04)	0.11 (0.02)	0.03 (0.01)	0.18 (0.06)
59	maaliol	1609	-	-	-	-	-
60	1,4-dimethylazulene	1616	10.08 (0.32)	40.95 (1.08)	42.32 (1.12)	28.05 (1.12)	19.55 (0.84)
61	(+)-spathulenol	1619	0.58 (0.12)	-	-	-	0.36 (0.10)
62	(-)-globulol	1631	0.79 (0.11)	0.22 (0.09)	0.23 (0.10)	0.13 (0.06)	0.09 (0.02)
63	220[M+](5) 132(100) 43(96)	1637	0.43 (0.12)	0.22 (0.08)	0.82 (0.12)	0.99 (0.22)	0.17 (0.04)
64	202[M+](3) 166(100) 165(98)	1645	-	-	-	-	0.01 (*)
65	202[M+](2)129(100) 172(72)	1655	0.82 (0.14)	1.80 (0.12)	1.75 (0.15)	2.97 (0.64)	1.36 (0.22)
66	200[M+](2) 145(100) 158(55)	1664	-	-	-	-	0.01 (*)
67	isospathulenol	1667	0.36 (0.11)	0.36 (0.09)	0.26 (0.03)	0.30 (0.08)	0.80 (0.12)
68	218[M+](2) 71(100) 57(93)	1674	0.39 (0.12)	0.40 (0.08)	0.11 (0.02)	0.02 (0.01)	0.18 (0.06)
69	220[M+](5) 129(100) 144(92)	1678	0.10 (0.02)	0.02 (0.01)	0.03 (0.01)	0.03 (0.01)	-
70	220[M+](31) 159(100) 105(81)	1684	0.25 (0.08)	0.33 (0.08)	0.34 (0.06)	0.51 (0.12)	0.85 (0.24)
71	202[M+](92) 143(100) 128(78)	1689	0.08 (0.02)	0.03 (0.01)	0.12 (0.02)	0.14 (0.03)	0.03 (0.01)
72	220[M+](11) 109(100) 121(51)	1695	0.16 (0.08)	0.08 (0.01)	0.09 (0.02)	0.04 (0.01)	0.10 (0.06)
73	220[M+](13) 159(100) 145(89)	1710	0.13 (0.06)	0.15 (0.03)	0.12 (0.02)	0.20 (0.04)	0.98 (0.12)
74	4-(cyclopent-1-enyl)benzoic acid methyl ester	1722	1.23 (0.21)	14.98 (2.22)	13.60 (0.32)	13.55 (0.42)	9.00 (0.33)
75	216[M+](10) 202(100) 159(96)	1744	0.29 (0.08)	0.12 (0.04)	0.02 (0.01)	0.09 (0.02)	0.12 (0.05)
76	216[M+](3) 202(100) 143(81)	1784	0.04 (0.01)	0.07 (0.02)	0.05 (0.01)	0.04 (0.01)	0.02 (0.01)
77	germacra-4(15),5,10(14)-trien-1-alpha-ol	1801	1.02 (0.10)	0.47 (0.10)	0.28 (0.03)	0.98 (0.11)	0.31 (0.10)
78	1,4-dimethyl-7-(1-methylethyl)-azulene	1823	0.15 (0.08)	0.08 (0.02)	0.08 (0.02)	0.01 (*)	0.08 (0.02)
79	methyl-2-methylazulene-1-carboxylate	2025	0.01 (*)	0.09 (0.03)	0.08 (0.02)	1.36 (0.14)	0.84 (0.12)
	Total		97.62 (13.58)	97.12 (8.08)	96.31 (8.61)	97.89 (11.53)	98.88 (11.96)
	% Identified		83.91 (9.93)	83.92 (6.37)	83.23 (5.71)	83.85 (8.24)	87.31 (9.70)
	Including:						
	Aliphatics		2.28 (0.64)	0.21 (0.05)	0.14 (0.04)	0.65 (0.14)	2.16 (0.41)
	Aromatics		1.31 (0.23)	15.38 (2.32)	13.88 (0.39)	13.83 (0.48)	9.08 (0.35)
	Monoterpene hydrocarbons		3.29 (0.53)	1.11 (0.14)	1.17 (0.24)	0.25 (0.08)	1.17 (0.31)
	Monoterpenoide hydrocarbons		0.46 (0.10)	1.45 (0.10)	1.28 (0.24)	1.21 (0.10)	0.64 (0.10)
	Sesquiterpene hydrocarbons		74.83 (8.09)	65.10 (3.55)	66.19 (4.65)	66.12 (7.16)	72.17 (8.17)
	Sesquiterpenoide hydrocarbons		1.74 (0.34)	0.67 (0.21)	0.57 (0.15)	1.79 (0.28)	2.09 (0.36)

-, * less than 0.01%. ^a^ Retention index on Quadex 007-5MS column. ^b^ For abbreviations of samples, see Table 1. ( ) standard deviation.

**Table 4:** Volatile compounds detected in the samples C-11–C-15.

No	Compounds	RI ^a^	Code ^b^				
			C-11	C-12	C-13	C-14	C-15
1	2-methyl-1-propanol	<700	-	-	-	0.01 (*)	-
2	pentanal	<700	0.02 (0.01)	0.06 (0.01)	0.09 (0.02)	0.01 (*)	0.07 (0.02)
3	cyclopentanol	748	-	-	-	0.07 (0.01)	-
4	3-methyl-1-butanol	768	0.01 (*)	0.02 (0.01)	0.02 (0.02)	0.06 (0.01)	0.01 (*)
5	hexanal	834	0.01 (*)	0.01 (*)	0.01 (*)	0.01 (*)	0.01 (*)
6	(*Z*)-3-hexen-1-ol	886	-	-	-	-	-
7	1-hexanol	896	0.10 (0.04)	0.09 (0.02)	0.35 (0.10)	0.22 (0.09)	0.28 (0.09)
8	106[M+](58) 91(100) 77(13)	902	0.28 (0.09)	0.31 (0.09)	0.10 (0.03)	-	0.11 (0.03)
9	tricyclene	939	-	-	-	0.01 (*)	-
10	α-pinene	952	0.08 (0.02)	0.18 (0.04)	0.08 (0.02)	0.04 (0.01)	0.15 (0.04)
11	camphene	971	-	-	-	0.01 (*)	-
12	β-pinene	1004	0.03 (0.01)	0.15 (0.04)	0.43 (0.10)	0.36 (0.09)	0.45 (0.10)
13	3-octanol	1018	0.02 (0.01)	0.01 (*)	0.07 (0.01)	0.11 (0.02)	0.03 (0.01)
14	limonene	1048	0.13 (0.06)	0.12 (0.04)	0.45 (0.09)	0.07 (0.01)	0.35 (0.09)
15	benzene acetaldehyde	1093	0.23 (0.09)	0.15 (0.06)	0.07 (0.01)	0.01 (*)	0.23 (0.08)
16	1-octen-3-yl-acetate	1123	0.38 (0.10)	0.33 (0.10)	0.60 (0.12)	1.42 (0.10)	0.48 (0.10)
17	benzene ethanol	1156	0.01 (*)	0.01 (*)	0.01 (*)	-	0.01 (*)
18	160[M+](49) 145(100) 117(34)	1224	3.47 (0.46)	2.95 (0.22)	1.28 (0.16)	2.19 (0.18)	1.47 (0.18)
19	β-cyclocitral	1257	0.79 (0.12)	1.26 (0.16)	1.64 (0.17)	0.52 (0.10)	0.79 (0.18)
20	1,2-dihydro-6-methylnaphthalene	1295	0.14 (0.04)	0.06 (0.01)	0.14 (0.04)	-	0.18 (0.04)
21	207[M]+(18) 121(100) 93(71)	1332	0.15 (0.04)	0.16 (0.04)	0.05 (0.01)	0.08 (0.02)	0.02 (0.01)
22	δ-elemene	1344	2.08 (0.87)	2.72 (0.76)	0.22 (0.09)	1.35 (0.12)	0.31 (0.09)
23	methylnaphthalene	1359	0.05 (0.01)	0.05 (0.01)	0.14 (0.04)	0.05 (0.01)	0.05 (0.01)
24	202[M+](18) 81(100) 96(83)	1364	0.18 (0.06)	0.20 (0.06)	0.13 (0.03)	0.11 (0.03)	0.14 (0.03)
25	anastreptene	1379	10.68 (2.46)	10.91 (2.42)	4.87 (0.86)	8.81 (1.32)	5.32 (0.98)
26	204[M+](25) 105(100) 161(83)	1382	0.28 (0.09)	0.58 (0.12)	0.10 (0.02)	0.11 (0.03)	0.11 (0.03)
27	202[M+](5) 159(100) 91(95)	1387	0.04 (0.01)	0.07 (0.01)	0.15 (0.04)	0.02 (0.01)	0.21 (0.06)
28	202[M+](5) 143(100) 128(92)	1392	1.09 (0.46)	1.24 (0.08)	0.89 (0.10)	0.62 (0.09)	1.09 (0.12)
29	β-elemene	1405	0.30 (0.08)	0.52 (0.10)	0.14 (0.04)	0.35 (0.10)	0.70 (0.22)
30	204[M]+(52) 161(100) 107(89)	1407	-	-	-	0.09 (0.02)	-
31	202[M]+(2) 143(100) 128(93)	1418	0.32 (0.10)	0.25 (0.09)	0.12 (0.04)	0.02 (0.01)	0.12 (0.03)
32	α-gurjunene	1423	0.25 (0.09)	0.19 (0.08)	0.16 (0.03)	0.16 (0.03)	0.11 (0.02)
33	202[M]+(2) 145(100) 160(35)	1426	0.46 (0.12)	0.30 (0.09)	0.12 (0.03)	0.22 (0.08)	0.21 (0.08)
34	β-gurjunene	1431	0.02 (0.01)	0.03 (0.01)	0.02 (0.01)	0.06 (0.01)	0.01 (*)
35	204[M]+(12) 159(100) 105(95)	1436	0.05 (0.01)	0.10 (0.03)	0.01 (*)	0.27 (0.08)	0.02 (0.01)
36	(-)-aristolene	1439	0.52 (0.14)	0.35 (0.10)	0.15 (0.04)	0.02 (0.01)	0.05 (0.01)
37	γ-gurjunene	1444	0.05 (0.01)	0.05 (0.01)	0.28 (0.09)	0.04 (0.01)	0.30 (0.08)
38	γ-maaliene	1449	1.08 (0.46)	0.85 (0.09)	0.14 (0.03)	0.62 (0.08)	0.32 (0.08)
39	α-maaliene	1455	0.28 (0.09)	0.45 (0.09)	0.31 (0.09)	0.51 (0.07)	0.25 (0.07)
40	alloaromadendrene	1459	4.01 (1.22)	4.96 (1.10)	1.13 (0.42)	4.98 (1.10)	1.05 (0.22)
41	selina-5,11-diene	1470	1.20 (0.12)	1.05 (0.12)	0.18 (0.05)	0.70 (0.09)	0.25 (0.08)
42	202[M]+(20) 105(100) 159(68)	1475	0.29 (0.08)	0.23 (0.08)	0.04 (0.01)	0.39 (0.08)	0.12 (0.04)
43	202[M+](30) 159(100) 131(75)	1479	0.37 (0.09)	0.25 (0.09)	0.37 (0.09)	0.50 (0.07)	0.46 (0.10)
44	dehydroaromadendrene	1482	1.04 (0.22)	1.33 (0.22)	-	0.41 (0.10)	-
45	204[M+](1) 142(100) 141(78)	1495	0.17 (0.06)	0.08 (0.01)	0.10 (0.02)	0.13 (0.03)	0.05 (0.01)
46	β-guaiene	1505	0.07 (0.01)	0.10 (0.04)	0.21 (0.09)	0.04 (0.01)	0.11 (0.02)
47	germacrene D	1510	2.57 (0.34)	2.60 (0.32)	1.15 (0.18)	2.98 (0.28)	1.56 (0.15)
48	ledene	1516	0.01 (*)	0.02 (0.01)	0.02 (0.01)	0.03 (0.01)	0.01 (*)
49	bicyclogermacrene	1521	10.95 (1.10)	10.47 (1.32)	1.67 (0.22)	8.73 (1.12)	1.45 (0.18)
50	220[M+](5) 148(100) 133(95)	1525	0.32 (0.09)	0.32 (0.09)	0.09 (0.02)	0.31 (0.09)	0.09 (0.02)
51	cuparene	1538	0.98 (0.10)	1.28 (0.21)	0.60 (0.09)	0.66 (0.08)	0.34 (0.09)
52	204[M+](22) 93(100) 105(68)	1543	0.10 (0.04)	0.12 (0.04)	0.04 (0.01)	0.11 (0.04)	0.03 (0.01)
53	202[M+](12) 157(100) 142(62)	1554	0.43 (0.12)	0.24 (0.09)	0.41 (0.10)	0.53 (0.12)	0.40 (0.10)
54	204[M+](18) 155(100) 119(82)	1558	0.10 (0.03)	0.03 (0.01)	0.16 (0.04)	0.02 (0.01)	0.24 (0.08)
55	200[M]+ (72) 143(100) 129(93)	1571	0.48 (0.10)	0.53 (0.10)	0.48 (0.10)	0.37 (0.09)	0.34 (0.09)
56	200[M+](73) 143(100) 157(97)	1579	0.19 (0.06)	0.15 (0.04)	0.05 (0.01)	0.12 (0.03)	0.04 (0.01)
57	204[M]+ (6) 69(100) 41(85)	1586	0.35 (0.10)	0.17 (0.06)	0.18 (0.05)	0.11 (0.03)	0.12 (0.02)
58	cadina-3,9-diene	1591	0.08 (0.02)	0.14 (0.04)	0.12 (0.03)	0.19 (0.04)	0.08 (0.01)
59	maaliol	1609	-	-	-	-	-
60	1,4-dimethylazulene	1616	27.03 (1.22)	26.17 (1.12)	58.79 (1.76)	30.28 (0.98)	54.28 (1.76)
61	(+)-spathulenol	1619	-	-	-	0.12 (0.04)	-
62	(−)-globulol	1631	0.18 (0.04)	0.25 (0.07)	0.47 (0.12)	0.54 (0.09)	0.12 (0.03)
63	220[M+](5) 132(100) 43(96)	1637	0.60 (0.14)	0.72 (0.14)	0.48 (0.12)	0.02 (0.01)	0.33 (0.09)
64	202[M+](3) 166(100) 165(98)	1645	-	-	-	0.03 (0.01)	-
65	202[M+](2)129(100) 172(72)	1655	3.72 (0.72)	2.98 (0.46)	1.07 (0.21)	1.92 (0.18)	1.72 (0.16)
66	200[M+](2) 145(100) 158(55)	1664	-	-	-	0.03 (0.01)	-
67	isospathulenol	1667	0.36 (0.09)	0.26 (0.09)	0.13 (0.03)	0.30 (0.08)	0.26 (0.07)
68	218[M+](2) 71(100) 57(93)	1674	0.04 (0.01)	0.11 (0.03)	-	0.02 (0.01)	-
69	220[M+](5) 129(100) 144(92)	1678	0.02 (0.01)	0.03 (0.01)	0.13 (0.04)	0.31 (0.14)	0.12 (0.03)
70	220[M+](31) 159(100) 105(81)	1684	0.39 (0.10)	0.38 (0.09)	0.16 (0.03)	4.22 (0.72)	0.29 (0.08)
71	202[M+](92) 143(100) 128(78)	1689	0.14 (0.04)	0.14 (0.03)	0.19 (0.03)	0.01 (*)	0.11 (0.02)
72	220[M+](11) 109(100) 121(51)	1695	0.06 (0.01)	0.12 (0.02)	-	0.09 (0.02)	-
73	220[M+](13) 159(100) 145(89)	1710	0.19 (0.04)	0.18 (0.04)	0.10 (0.02)	1.43 (0.12)	0.11 (0.02)
74	4-(cyclopent-1-enyl)benzoic acid methyl ester	1722	15.87 (0.48)	14.50 (0.52)	14.55 (0.56)	16.10 (0.62)	16.92 (0.64)
75	216[M+](10) 202(100) 159(96)	1744	0.13 (0.04)	0.04 (0.01)	0.02 (0.01)	0.05 (0.01)	0.08 (0.02)
76	216[M+](3) 202(100) 143(81)	1784	0.06 (0.01)	0.03 (0.01)	0.03 (0.01)	0.01 (*)	0.06 (0.01)
77	germacra-4(15),5,10(14)-trien-1-alpha-ol	1801	0.70 (0.09)	0.72 (0.08)	0.57 (0.08)	0.35 (0.09)	0.52 (0.09)
78	1,4-dimethyl-7-(1-methylethyl)-azulene	1823	0.06 (0.01)	0.02 (0.01)	0.05 (0.01)	0.01 (*)	0.11 (0.02)
79	methyl-2-methylazulene-1-carboxylate	2025	1.22 (0.16)	1.44 (0.14)	1.52 (0.14)	1.62 (0.12)	1.38 (0.12)
	Total		98.06 (13.27)	96.89 (11.84)	98.60 (7.19)	97.39 (9.41)	97.11 (7.28)
	% Identified		83.59 (9.94)	83.88 (9.56)	91.55 (5.82)	82.94 (7.05)	88.90 (5.79)
	Including:						
	Aliphatics		0.54 (0.16)	0.52 (0.14)	1.14 (0.27)	1.91 (0.23)	0.88 (0.22)
	Aromatics		16.30 (0.62)	14.77 (0.60)	14.91 (0.65)	16.16 (0.63)	17.39 (0.77)
	Monoterpene hydrocarbons		0.24 (0.09)	0.45 (0.12)	0.96 (0.21)	0.49 (0.11)	0.95 (0.23)
	Monoterpenoide hydrocarbons		0.79 (0.12)	1.26 (0.16)	1.64 (0.17)	0.52 (0.10)	0.79 (0.18)
	Sesquiterpene hydrocarbons		63.96 (8.66)	64.93 (8.24)	70.78 (4.22)	61.28 (5.65)	67.13 (4.17)
	Sesquiterpenoide hydrocarbons		1.76 (0.29)	1.95 (0.30)	2.12 (0.30)	2.58 (0.33)	1.76 (0.22)

-, * less than 0.01%. ^a^ Retention index on Quadex 007-5MS column. ^b^ For abbreviations of samples, see Table 1. ( ) standard deviation.

**Table 5:** Volatile compounds detected in the samples C-16–C-20.

No	Compounds	RI ^a^	Code ^b^				
			C-16	C-17	C-18	C-19	C-20
1	2-methyl-1-propanol	<700	-	-	-	0.11 (0.04)	-
2	pentanal	<700	0.09 (0.02)	0.01 (*)	0.11 (0.04)	0.07 (0.01)	0.09 (0.02)
3	cyclopentanol	748	-	-	-	0.06 (0.01)	-
4	3-methyl-1-butanol	768	0.02 (0.01)	0.02 (0.01)	0.07 (0.01)	0.38 (0.09)	0.05 (0.01)
5	hexanal	834	0.01 (*)	0.01 (*)	0.03 (0.01)	0.33 (0.08)	0.01 (*)
6	(*Z*)-3-hexen-1-ol	886	-	-	-	0.19 (0.05)	-
7	1-hexanol	896	0.41 (0.09)	0.08 (0.02)	-	0.11 (0.03)	-
8	106[M+](58) 91(100) 77(13)	902	0.08 (0.02)	0.57 (0.09)	0.68 (0.10)	1.25 (0.12)	0.59 (0.11)
9	tricyclene	939	-	-	-	0.08 (0.02)	-
10	α-pinene	952	0.13 (0.03)	0.05 (0.01)	0.04 (0.01)	0.38 (0.09)	0.07 (0.02)
11	camphene	971	-	-	-	0.68 (0.11)	-
12	β-pinene	1004	0.32 (0.09)	0.05 (0.01)	0.73 (0.12)	0.27 (0.08)	0.65 (0.11)
13	3-octanol	1018	0.04 (0.01)	0.02 (0.01)	-	0.15 (0.04)	-
14	limonene	1048	0.15 (0.04)	0.13 (0.03)	0.61 (0.10)	1.79 (0.14)	0.47 (0.09)
15	benzene acetaldehyde	1093	0.15 (0.03)	0.07 (0.01)	0.15 (0.02)	0.11 (0.03)	0.25 (0.05)
16	1-octen-3-yl-acetate	1123	0.23 (0.08)	0.45 (0.09)	0.03 (0.01)	1.01 (0.10)	0.01 (*)
17	benzene ethanol	1156	0.01 (*)	0.01 (*)	0.02 (0.01)	-	0.02 (0.01)
18	160[M+](49) 145(100) 117(34)	1224	1.95 (0.18)	3.10 (0.42)	4.29 (0.26)	2.29 (0.22)	3.39 (0.22)
19	β-cyclocitral	1257	0.76 (0.18)	1.11 (0.10)	1.37 (0.12)	0.51 (0.10)	1.38 (0.26)
20	1,2-dihydro-6-methylnaphthalene	1295	0.16 (0.03)	0.12 (0.03)	0.16 (0.04)	-	0.11 (0.04)
21	207[M]+(18) 121(100) 93(71)	1332	0.04 (0.01)	0.10 (0.02)	0.05 (0.01)	0.08 (0.02)	0.08 (0.02)
22	δ-elemene	1344	0.21 (0.08)	2.01 (0.26)	0.71 (0.10)	1.23 (0.12)	0.61 (0.11)
23	methylnaphthalene	1359	0.05 (0.01)	0.06 (0.01)	0.17 (0.04)	0.03 (0.01)	0.11 (0.03)
24	202[M+](18) 81(100) 96(83)	1364	0.11 (0.02)	0.29 (0.06)	0.11 (0.03)	0.28 (0.08)	0.10 (0.02)
25	anastreptene	1379	4.91 (0.78)	11.48 (0.82)	6.68 (0.82)	31.01 (2.12)	6.95 (0.28)
26	204[M+](25) 105(100) 161(83)	1382	0.17 (0.04)	0.34 (0.07)	0.48 (0.10)	-	0.47 (0.09)
27	202[M+](5) 159(100) 91(95)	1387	0.18 (0.04)	0.06 (0.01)	0.13 (0.04)	-	0.21 (0.06)
28	202[M+](5) 143(100) 128(92)	1392	1.11 (0.11)	1.06 (0.10)	1.81 (0.14)	0.52 (0.12)	1.78 (0.12)
29	β-elemene	1405	0.15 (0.04)	0.61 (0.10)	0.21 (0.07)	0.41 (0.10)	0.22 (0.04)
30	204[M]+(52) 161(100) 107(89)	1407	-	-	-	0.02 (0.01)	-
31	202[M]+(2) 143(100) 128(93)	1418	0.14 (0.03)	0.13 (0.03)	0.31 (0.09)	0.21 (0.06)	0.41 (0.09)
32	α-gurjunene	1423	0.12 (0.03)	0.25 (0.04)	0.18 (0.05)	0.59 (0.12)	0.18 (0.05)
33	202[M]+(2) 145(100) 160(35)	1426	0.24 (0.07)	0.27 (0.04)	0.17 (0.04)	0.03 (0.01)	0.37 (0.08)
34	β-gurjunene	1431	0.02 (0.01)	0.05 (0.01)	0.19 (0.04)	0.48 (0.10)	0.17 (0.04)
35	204[M]+(12) 159(100) 105(95)	1436	0.05 (0.01)	0.02 (0.01)	0.12 (0.03)	0.21 (0.06)	0.15 (0.04)
36	(-)-aristolene	1439	0.08 (0.02)	0.47 (0.09)	0.33 (0.09)	0.33 (0.08)	0.27 (0.08)
37	γ-gurjunene	1444	0.32 (0.09)	0.07 (0.01)	0.02 (0.01)	0.18 (0.04)	0.03 (0.01)
38	γ-maaliene	1449	0.25 (0.08)	0.72 (0.10)	0.34 (0.09)	0.87 (0.12)	0.87 (0.10)
39	α-maaliene	1455	0.22 (0.08)	0.39 (0.09)	0.37 (0.09)	0.78 (0.11)	0.35 (0.09)
40	alloaromadendrene	1459	1.92 (0.21)	5.11 (0.76)	3.59 (0.26)	8.91 (1.56)	3.28 (1.10)
41	selina-5,11-diene	1470	0.30 (0.09)	0.79 (0.10)	0.59 (0.12)	1.11 (0.14)	0.91 (0.14)
42	202[M]+(20) 105(100) 159(68)	1475	0.14 (0.03)	0.19 (0.04)	0.67 (0.11)	1.47 (0.16)	0.20 (0.06)
43	202[M+](30) 159(100) 131(75)	1479	0.51 (0.12)	0.33 (0.09)	0.47 (0.10)	2.18 (0.11)	0.59 (0.10)
44	dehydroaromadendrene	1482	-	1.33 (0.14)	0.54 (0.12)	1.41 (0.14)	0.57 (0.10)
45	204[M+](1) 142(100) 141(78)	1495	0.05 (0.01)	0.08 (0.02)	0.11 (0.03)	0.18 (0.04)	0.21 (0.04)
46	β-guaiene	1505	0.15 (0.03)	0.19 (0.04)	0.05 (0.01)	0.07 (0.01)	0.03 (0.01)
47	germacrene D	1510	1.86 (0.18)	3.08 (0.32)	2.76 (0.16)	5.18 (0.32)	3.51 (1.10)
48	ledene	1516	0.02 (0.01)	0.15 (0.04)	0.06 (0.02)	0.15 (0.03)	-
49	bicyclogermacrene	1521	1.78 (0.15)	10.35 (0.76)	4.11 (0.22)	9.01 (1.86)	4.47 (1.21)
50	220[M+](5) 148(100) 133(95)	1525	0.11 (0.02)	0.61 (0.12)	0.15 (0.03)	0.12 (0.04)	0.21 (0.05)
51	cuparene	1538	0.42 (0.10)	1.88 (0.22)	0.63 (0.10)	1.96 (0.21)	0.81 (0.12)
52	204[M+](22) 93(100) 105(68)	1543	0.05 (0.01)	0.09 (0.02)	0.05 (0.01)	0.02 (0.01)	0.11 (0.03)
53	202[M+](12) 157(100) 142(62)	1554	0.20 (0.06)	0.54 (0.09)	0.33 (0.12)	1.28 (0.18)	0.35 (0.09)
54	204[M+](18) 155(100) 119(82)	1558	0.27 (0.08)	0.04 (0.01)	0.07 (0.02)	0.08 (0.02)	0.07 (0.02)
55	200[M]+ (72) 143(100) 129(93)	1571	0.38 (0.09)	0.64 (0.10)	0.18 (0.04)	0.38 (0.09)	0.35 (0.08)
56	200[M+](73) 143(100) 157(97)	1579	0.02 (0.01)	0.29 (0.04)	0.07 (0.01)	0.21 (0.06)	0.05 (0.01)
57	204[M]+ (6) 69(100) 41(85)	1586	0.15 (0.03)	0.20 (0.04)	0.18 (0.04)	0.05 (0.01)	0.27 (0.06)
58	cadina-3,9-diene	1591	0.07 (0.01)	0.15 (0.03)	0.23 (0.05)	0.13 (0.03)	0.17 (0.03)
59	maaliol	1609	-	-	-	-	-
60	1,4-dimethylazulene	1616	56.13 (1.86)	29.05 (0.88)	45.98 (2.46)	10.15 (0.76)	40.87 (1.52)
61	(+)-spathulenol	1619	-	-	-	0.51 (0.10)	-
62	(-)-globulol	1631	0.23 (0.06)	0.23 (0.05)	0.47 (0.10)	0.72 (0.10)	0.25 (0.06)
63	220[M+](5) 132(100) 43(96)	1637	0.42 (0.11)	0.97 (0.10)	0.52 (0.11)	0.39 (0.09)	0.18 (0.04)
64	202[M+](3) 166(100) 165(98)	1645	-	-	-	-	-
65	202[M+](2)129(100) 172(72)	1655	1.22 (0.13)	2.61 (0.28)	1.59 (0.16)	0.79 (0.11)	1.91 (0.16)
66	200[M+](2) 145(100) 158(55)	1664	-	-	-	-	-
67	isospathulenol	1667	0.20 (0.06)	0.41 (0.09)	0.29 (0.07)	0.41 (0.09)	0.41 (0.09)
68	218[M+](2) 71(100) 57(93)	1674	-	0.03 (0.01)	0.11 (0.03)	0.40 (0.09)	0.39 (0.08)
69	220[M+](5) 129(100) 144(92)	1678	0.11 (0.03)	0.07 (0.01)	0.08 (0.02)	0.11 (0.03)	0.02 (0.01)
70	220[M+](31) 159(100) 105(81)	1684	0.28 (0.08)	0.47 (0.09)	0.51 (0.10)	0.18 (0.04)	0.35 (0.08)
71	202[M+](92) 143(100) 128(78)	1689	0.17 (0.03)	0.09 (0.01)	0.15 (0.03)	0.09 (0.02)	0.05 (0.01)
72	220[M+](11) 109(100) 121(51)	1695	-	0.07 (0.01)	0.08 (0.02)	0.16 (0.03)	0.09 (0.02)
73	220[M+](13) 159(100) 145(89)	1710	0.10 (0.03)	0.14 (0.03)	0.13 (0.03)	0.15 (0.04)	0.13 (0.03)
74	4-(cyclopent-1-enyl)benzoic acid methyl ester	1722	15.22 (0.68)	9.82 (0.52)	13.55 (0.68)	1.17 (0.18)	15.87 (0.70)
75	216[M+](10) 202(100) 159(96)	1744	0.02 (0.01)	0.11 (0.03)	0.11 (0.03)	0.29 (0.08)	0.14 (0.03)
76	216[M+](3) 202(100) 143(81)	1784	0.03 (0.01)	0.05 (0.01)	0.07 (0.01)	0.03 (0.01)	0.08 (0.01)
77	germacra-4(15),5,10(14)-trien-1-alpha-ol	1801	0.48 (0.09)	1.26 (0.18)	0.38 (0.09)	1.05 (0.14)	0.45 (0.10)
78	1,4-dimethyl-7-(1-methylethyl)-azulene	1823	0.11 (0.03)	0.26 (0.04)	0.06 (0.01)	0.16 (0.05)	0.07 (0.02)
79	methyl-2-methylazulene-1-carboxylate	2025	1.21 (0.13)	1.50 (0.16)	0.09 (0.02)	0.03 (0.01)	0.15 (0.03)
	Total		97.21 (6.92)	97.36 (8.18)	99.64 (8.27)	97.72 (11.63)	97.99 (9.63)
	% Identified		88.91 (5.52)	83.80 (6.18)	85.86 (6.38)	84.27 (9.67)	84.69 (7.77)
	Including:						
	Aliphatics		0.80 (0.21)	0.59 (0.13)	0.24 (0.07)	2.41 (0.45)	0.16 (0.03)
	Aromatics		15.59 (0.75)	10.08 (0.57)	14.01 (0.79)	1.31 (0.22)	16.36 (0.83)
	Monoterpene hydrocarbons		0.60 (0.16)	0.23 (0.05)	1.38 (0.23)	3.20 (0.44)	1.19 (0.22)
	Monoterpenoide hydrocarbons		0.76 (0.18)	1.11 (0.10)	1.37 (0.12)	0.51 (0.10)	1.38 (0.26)
	Sesquiterpene hydrocarbons		69.52 (3.97)	69.65 (5.03)	68.01 (4.98)	75.17 (8.16)	64.79 (6.25)
	Sesquiterpenoide hydrocarbons		1.64 (0.25)	2.14 (0.30)	0.85 (0.19)	1.67 (0.30)	0.81 (0.18)

-, * less than 0.01%. ^a^ Retention index on Quadex 007-5MS column. ^b^ For abbreviations of samples, see Table 1. ( ) standard deviation.

**Table 6:** Volatile compounds detected in the samples C-21–C-25.

No.	Compounds	RI ^a^	Code ^b^				
			C-21	C-22	C-23	C-24	C-25
1	2-methyl-1-propanol	<700	-	-	0.11 (0.03)	-	-
2	pentanal	<700	0.07 (0.02)	0.03 (0.01)	0.04 (0.01)	0.06 (0.01)	0.10 (0.02)
3	cyclopentanol	748	-	-	0.03 (0.01)	-	-
4	3-methyl-1-butanol	768	0.05 (0.01)	0.01 (*)	0.19 (0.05)	0.02 (0.01)	0.05 (0.01)
5	hexanal	834	0.02 (0.01)	0.05 (0.01)	0.16 (0.04)	0.02 (0.01)	0.03 (0.01)
6	(*Z*)-3-hexen-1-ol	886	-	-	0.13 (0.03)	-	-
7	1-hexanol	896	-	0.08 (0.02)	0.15 (0.04)	0.12 (0.03)	0.11 (0.03)
8	106[M+](58) 91(100) 77(13)	902	0.59 (0.12)	0.45 (0.10)	0.57 (0.10)	0.25 (0.04)	0.25 (0.04)
9	tricyclene	939	-	-	0.07 (0.02)	-	-
10	α-pinene	952	0.11 (0.03)	0.07 (0.01)	0.21 (0.03)	0.11 (0.02)	0.23 (0.04)
11	camphene	971	-	-	0.52 (0.10)	-	-
12	β-pinene	1004	0.49 (0.10)	0.05 (0.01)	0.16 (0.03)	0.05 (0.01)	0.13 (0.03)
13	3-octanol	1018	-	0.01 (*)	0.25 (0.04)	0.03 (0.01)	0.01 (*)
14	limonene	1048	0.58 (0.11)	0.20 (0.05)	0.26 (0.04)	0.15 (0.04)	0.15 (0.02)
15	benzene acetaldehyde	1093	0.17 (0.03)	0.09 (0.02)	0.07 (0.02)	0.27 (0.04)	0.14 (0.02)
16	1-octen-3-yl-acetate	1123	0.06 (0.01)	0.51 (0.10)	1.18 (0.12)	0.31 (0.05)	0.39 (0.09)
17	benzene ethanol	1156	0.02 (0.01)	0.03 (0.01)	-	0.01 (*)	0.01 (*)
18	160[M+](49) 145(100) 117(34)	1224	4.95 (0.18)	3.52 (0.14)	2.26 (0.18)	3.52 (0.22)	3.02 (0.22)
19	β-cyclocitral	1257	1.31 (0.26)	1.27 (0.18)	0.67 (0.10)	0.81 (0.12)	1.31 (0.18)
20	1,2-dihydro-6-methylnaphthalene	1295	0.06 (0.01)	0.13 (0.03)	-	0.14 (0.04)	0.05 (0.01)
21	207[M]+(18) 121(100) 93(71)	1332	0.11 (0.02)	0.15 (0.04)	0.11 (0.03)	0.17 (0.03)	0.17 (0.02)
22	δ-elemene	1344	0.38 (0.09)	2.29 (0.22)	1.24 (0.14)	2.12 (0.22)	2.83 (0.16)
23	methylnaphthalene	1359	0.11 (0.03)	0.11 (0.03)	0.09 (0.02)	0.06 (0.01)	0.07 (0.02)
24	202[M+](18) 81(100) 96(83)	1364	0.15 (0.04)	0.21 (0.04)	0.11 (0.03)	0.18 (0.04)	0.15 (0.02)
25	anastreptene	1379	6.79 (0.28)	12.01 (0.34)	20.89 (0.56)	10.57 (0.34)	11.05 (0.34)
26	204[M+](25) 105(100) 161(83)	1382	0.52 (0.10)	0.35 (0.09)	0.05 (0.01)	0.31 (0.08)	0.61 (0.12)
27	202[M+](5) 159(100) 91(95)	1387	0.06 (0.01)	0.05 (0.01)	-	0.06 (0.02)	0.11 (0.03)
28	202[M+](5) 143(100) 128(92)	1392	1.38 (0.12)	1.25 (0.17)	0.61 (0.10)	1.11 (0.14)	1.31 (0.16)
29	β-elemene	1405	0.55 (0.10)	0.47 (0.09)	0.39 (0.09)	0.32 (0.09)	0.59 (0.10)
30	204[M]+(52) 161(100) 107(89)	1407	-	-	0.05 (0.01)	-	-
31	202[M]+(2) 143(100) 128(93)	1418	0.20 (0.04)	0.18 (0.04)	0.11 (0.03)	0.27 (0.08)	0.31 (0.09)
32	α-gurjunene	1423	0.15 (0.02)	0.23 (0.06)	0.29 (0.06)	0.26 (0.07)	0.22 (0.07)
33	202[M]+(2) 145(100) 160(35)	1426	0.23 (0.04)	0.21 (0.05)	0.13 (0.03)	0.51 (0.11)	0.33 (0.08)
34	β-gurjunene	1431	0.16 (0.03)	0.06 (0.02)	0.09 (0.02)	0.03 (0.01)	0.09 (0.02)
35	204[M]+(12) 159(100) 105(95)	1436	0.14 (0.02)	0.05 (0.01)	0.28 (0.04)	0.03 (0.01)	0.15 (0.03)
36	(-)-aristolene	1439	0.22 (0.04)	0.41 (0.09)	0.17 (0.04)	0.49 (0.10)	0.28 (0.08)
37	γ-gurjunene	1444	0.05 (0.01)	0.04 (0.01)	0.13 (0.03)	0.05 (0.01)	0.09 (0.02)
38	γ-maaliene	1449	0.81 (0.12)	0.58 (0.10)	0.69 (0.10)	1.17 (0.16)	0.93 (0.12)
39	α-maaliene	1455	0.52 (0.09)	0.39 (0.09)	0.59 (0.11)	0.24 (0.05)	0.51 (0.10)
40	alloaromadendrene	1459	4.28 (1.12)	4.69 (0.32)	6.32 (1.18)	4.15 (0.32)	5.21 (0.22)
41	selina-5,11-diene	1470	0.89 (0.12)	0.83 (0.12)	0.27 (0.05)	0.98 (0.18)	1.21 (0.21)
42	202[M]+(20) 105(100) 159(68)	1475	0.19 (0.05)	0.17 (0.04)	0.92 (0.17)	0.33 (0.09)	0.33 (0.09)
43	202[M+](30) 159(100) 131(75)	1479	0.21 (0.04)	0.22 (0.05)	1.04 (0.16)	0.41 (0.09)	0.23 (0.06)
44	dehydroaromadendrene	1482	0.49 (0.10)	1.09 (0.12)	0.92 (0.12)	1.09 (0.22)	1.15 (0.18)
45	204[M+](1) 142(100) 141(78)	1495	0.09 (0.02)	0.16 (0.04)	0.31 (0.07)	0.25 (0.04)	0.09 (0.02)
46	β-guaiene	1505	0.04 (0.01)	0.14 (0.04)	0.24 (0.04)	0.05 (0.01)	0.19 (0.04)
47	germacrene D	1510	3.15 (0.11)	2.69 (0.160	3.25 (0.18)	2.63 (0.18)	2.47 (0.18)
48	ledene	1516	-	0.06 (0.01)	0.03 (0.01)	0.01 (*)	0.02 (0.01)
49	bicyclogermacrene	1521	4.39 (1.20)	9.65 (2.22)	15.28 (1.42)	11.18 (1.11)	10.25 (1.02)
50	220[M+](5) 148(100) 133(95)	1525	0.18 (0.05)	0.61 (0.10)	0.51 (0.11)	0.28 (0.06)	0.39 (0.09)
51	cuparene	1538	0.71 (0.10)	1.53 (0.13)	1.12 (0.14)	1.05 (0.16)	1.21 (0.14)
52	204[M+](22) 93(100) 105(68)	1543	0.11 (0.03)	0.07 (0.02)	0.08 (0.01)	0.08 (0.02)	0.19 (0.04)
53	202[M+](12) 157(100) 142(62)	1554	0.21 (0.04)	0.51 (0.10)	0.24 (0.04)	0.37 (0.09)	0.18 (0.03)
54	204[M+](18) 155(100) 119(82)	1558	0.01 (*)	0.05 (0.01)	0.06 (0.01)	0.10 (0.02)	0.01 (*)
55	200[M]+ (72) 143(100) 129(93)	1571	0.28 (0.08)	0.81 (0.11)	0.41 (0.09)	0.51 (0.09)	0.64 (0.12)
56	200[M+](73) 143(100) 157(97)	1579	0.06 (0.02)	0.26 (0.04)	0.15 (0.03)	0.21 (0.05)	0.15 (0.03)
57	204[M]+ (6) 69(100) 41(85)	1586	0.11 (0.04)	0.19 (0.04)	0.11 (0.02)	0.42 (0.09)	0.21 (0.05)
58	cadina-3,9-diene	1591	0.13 (0.04)	0.06 (0.01)	0.21 (0.05)	0.09 (0.02)	0.15 (0.03)
59	maaliol	1609	-	-	-	-	-
60	1,4-dimethylazulene	1616	41.89 (1.62)	28.09 (0.88)	20.13 (0.86)	26.99 (0.92)	26.29 (0.92)
61	(+)-spathulenol	1619	-	-	0.41 (0.09)	-	-
62	(−)-globulol	1631	0.23 (0.06)	0.17 (0.04)	0.04 (0.01)	0.21 (0.04)	0.18 (0.03)
63	220[M+](5) 132(100) 43(96)	1637	0.87 (0.10)	1.09 (0.16)	0.15 (0.02)	0.57 (0.10)	0.82 (0.12)
64	202[M+](3) 166(100) 165(98)	1645	-	-	0.01 (*)	-	-
65	202[M+](2)129(100) 172(72)	1655	1.75 (0.16)	2.97 (0.18)	1.15 (0.22)	3.84 (0.18)	3.12 (0.22)
66	200[M+](2) 145(100) 158(55)	1664	-	-	0.01 (*)	-	-
67	isospathulenol	1667	0.31 (0.08)	0.27 (0.05)	0.69 (0.12)	0.43 (0.09)	0.31 (0.09)
68	218[M+](2) 71(100) 57(93)	1674	0.15 (0.03)	0.05 (0.01)	0.19 (0.04)	0.02 (0.01)	0.11 (0.03)
69	220[M+](5) 129(100) 144(92)	1678	0.03 (0.01)	0.07 (0.02)	-	0.02 (0.01)	0.03 (0.01)
70	220[M+](31) 159(100) 105(81)	1684	0.41 (0.09)	0.57 (0.10)	0.95 (0.12)	0.42 (0.09)	0.41 (0.09)
71	202[M+](92) 143(100) 128(78)	1689	0.13 (0.03)	0.19 (0.04)	0.05 (0.01)	0.15 (0.03)	0.21 (0.04)
72	220[M+](11) 109(100) 121(51)	1695	0.08 (0.01)	0.90 (0.12)	0.09 (0.02)	0.06 (0.01)	0.15 (0.04)
73	220[M+](13) 159(100) 145(89)	1710	0.15 (0.02)	0.18 (0.040	0.98 (0.18)	0.21 (0.03)	0.16 (0.03)
74	4-(cyclopent-1-enyl)benzoic acid methyl ester	1722	14.54 (0.78)	12.95 (0.68)	8.15 (0.56)	15.92 (0.34)	15.02 (0.67)
75	216[M+](10) 202(100) 159(96)	1744	0.02 (0.01)	0.07 (0.02)	0.15 (0.04)	0.15 (0.03)	0.04 (0.01)
76	216[M+](3) 202(100) 143(81)	1784	0.07 (0.01)	0.09 (0.02)	0.02 (0.01)	0.05 (0.01)	0.03 (0.01)
77	germacra-4(15),5,10(14)-trien-1-alpha-ol	1801	0.31 (0.10)	1.09 (0.14)	0.35 (0.09)	0.69 (0.12)	0.69 (0.10)
78	1,4-dimethyl-7-(1-methylethyl)-azulene	1823	0.08 (0.01)	0.03 (0.01)	0.06 (0.01)	0.06 (0.01)	0.02 (0.01)
79	methyl-2-methylazulene-1-carboxylate	2025	0.11 (0.03)	1.41 (0.12)	0.91 (0.10)	1.29 (0.12)	1.38 (0.11)
	Total		97.67 (9.41)	99.52 (8.50)	99.01 (8.84)	99.09 (7.22)	99.03 (7.39)
	% Identified		84.23 (7.88)	83.87 (6.55)	87.15 (6.91)	84.23 (5.31)	85.12 (5.45)
	Including:						
	Aliphatics		0.20 (0.05)	0.69 (0.14)	2.24 (0.37)	0.56 (0.12)	0.69 (0.16)
	Aromatics		14.90 (0.86)	13.31 (0.77)	8.31 (0.60)	16.40 (0.43)	15.29 (0.72)
	Monoterpene hydrocarbons		1.18 (0.24)	0.32 (0.07)	1.22 (0.22)	0.31 (0.07)	0.51 (0.09)
	Monoterpenoide hydrocarbons		1.31 (0.26)	1.27 (0.18)	0.67 (0.10)	0.81 (0.12)	1.31 (0.18)
	Sesquiterpene hydrocarbons		65.99 (6.30)	66.43 (5.18)	72.66 (5.30)	64.22 (4.30)	65.45 (4.07)
	Sesquiterpenoide hydrocarbons		0.65 (0.17)	1.85 (0.21)	2.05 (0.32)	1.93 (0.27)	1.87 (0.23)

-, * less than 0.01%. ^a^ Retention index on Quadex 007-5MS column. ^b^ For abbreviations of samples, see Table 1. ( ) standard deviation.

**Table 7:** Volatile compounds detected in the samples C-26–C-30.

No.	Compounds	RI ^a^	Code ^b^				
			C-26	C-27	C-28	C-29	C-30
1	2-methyl-1-propanol	<700	-	0.01 (*)	-	-	-
2	pentanal	<700	0.07 (0.02)	0.01 (*)	0.02 (0.01)	0.12 (0.03)	0.03 (0.01)
3	cyclopentanol	748	-	0.05 (0.01)	-	-	-
4	3-methyl-1-butanol	768	0.05 (0.05)	0.05 (0.01)	0.01 (*)	0.01 (*)	0.03 (0.01)
5	hexanal	834	0.01 (*)	0.01 (*)	0.01 (*)	0.01 (*)	0.03 (0.01)
6	(*Z*)-3-hexen-1-ol	886	-	-	-	-	-
7	1-hexanol	896	0.29 (0.04)	0.18 (0.03)	0.15 (0.02)	0.23 (0.04)	0.12 (0.03)
8	106[M+](58) 91(100) 77(13)	902	0.15 (0.03)	-	0.21 (0.04)	0.15 (0.04)	0.48 (0.09)
9	tricyclene	939	-	0.01 (*)	-	-	-
10	α-pinene	952	0.11 (0.02)	0.03 (0.01)	0.19 (0.03)	0.15 ((0.04)	0.06 (0.01)
11	camphene	971	-	0.02 (0.01)	-	-	-
12	β-pinene	1004	0.51 (0.09)	0.28 (0.04)	0.33 (0.06)	0.42 (0.09)	0.07 (0.01)
13	3-octanol	1018	0.06 (0.01)	0.09 (0.02)	0.10 (0.02)	0.01 (*)	0.03 (0.01)
14	limonene	1048	0.41 (0.09)	0.11 (0.03)	0.42 (0.09)	0.21 (0.04)	0.11 (0.03)
15	benzene acetaldehyde	1093	0.07 (0.01)	0.01 (*)	0.16 (0.04)	0.11 (0.03)	0.04 (0.01)
16	1-octen-3-yl-acetate	1123	0.59 (0.09)	1.38 (0.12)	0.55 (0.09)	0.33 (0.07)	0.62 (0.10)
17	benzene ethanol	1156	0.01 (*)	-	0.01 (*)	0.01 (*)	0.02 (0.01)
18	160[M+](49) 145(100) 117(34)	1224	1.31 (0.14)	2.25 (0.18)	1.38 (0.16)	1.73 (0.32)	3.38 (0.34)
19	β-cyclocitral	1257	1.59 (0.11)	0.47 (0.09)	0.85 (0.12)	0.69 (0.09)	1.19 (0.14)
20	1,2-dihydro-6-methylnaphthalene	1295	0.19 (0.03)	-	0.21 (0.03)	0.11 (0.03)	0.08 (0.01)
21	207[M]+(18) 121(100) 93(71)	1332	0.06 (0.01)	0.05 (0.01)	0.01 (*)	0.01 (*)	0.13 (0.03)
22	δ-elemene	1344	0.25 (0.03)	1.41 (0.12)	0.24 (0.04)	0.17 (0.04)	2.23 (0.32)
23	methylnaphthalene	1359	0.14 (0.02)	0.04 (0.01)	0.01 (*)	0.05 (0.01)	0.08 (0.01)
24	202[M+](18) 81(100) 96(83)	1364	0.15 (0.03)	0.11 (0.02)	0.22 (0.04)	0.06 (0.01)	0.18 (0.04)
25	anastreptene	1379	5.06 (0.32)	8.93 (0.84)	4.98 (0.34)	5.23 (0.35)	12.85 (0.54)
26	204[M+](25) 105(100) 161(83)	1382	0.12 (0.04)	0.12 (0.03)	0.21 (0.05)	0.13 (0.03)	0.32 (0.09)
27	202[M+](5) 159(100) 91(95)	1387	0.17 (0.03)	0.03 (0.01)	0.17 (0.03)	0.22 (0.04)	0.03 (0.01)
28	202[M+](5) 143(100) 128(92)	1392	0.91 (0.10)	0.65 (0.11)	1.15 (0.14)	1.01 (0.22)	1.18 (0.12)
29	β-elemene	1405	0.17 (0.03)	0.28 (0.06)	0.57 (0.09)	0.10 (0.02)	0.74 (0.10)
30	204[M]+(52) 161(100) 107(89)	1407	-	0.05 (0.01)	-	-	-
31	202[M]+(2) 143(100) 128(93)	1418	0.15 (0.02)	0.01 (*)	0.07 (0.02)	0.14 (0.03)	0.23 (0.04)
32	α-gurjunene	1423	0.17 (0.02)	0.11 (0.03)	0.11 (0.03)	0.06 (0.01)	0.23 (0.04)
33	202[M]+(2) 145(100) 160(35)	1426	0.15 (0.02)	0.18 (0.03)	0.18 (0.04)	0.33 (0.08)	0.27 (0.05)
34	β-gurjunene	1431	0.05 (0.01)	0.03 (0.01)	0.01 (*)	0.01 (*)	0.04 (0.01)
35	204[M]+(12) 159(100) 105(95)	1436	0.02 (0.01)	0.19 (0.04)	0.01 (*)	0.02 (0.01)	0.04 (0.01)
36	(−)-aristolene	1439	0.17 (0.03)	0.01 (*)	0.02 (0.01)	0.05 (0.01)	0.24 (0.05)
37	γ-gurjunene	1444	0.31 (0.08)	0.01 (*)	0.35 (0.07)	0.43 (0.09)	0.07 (0.01)
38	γ-maaliene	1449	0.19 (0.03)	0.47 (0.09)	0.27 (0.05)	0.17 (0.03)	0.55 (0.09)
39	α-maaliene	1455	0.35 (0.08)	0.52 (0.09)	0.25 (0.04)	0.22 (0.04)	0.44 (0.09)
40	alloaromadendrene	1459	1.03 (0.18)	5.21 (0.18)	1.15 (0.14)	2.03 (0.32)	4.66 (0.36)
41	selina-5,11-diene	1470	0.25 (0.03)	0.61 (0.10)	0.15 (0.03)	0.34 (0.06)	0.82 (0.10)
42	202[M]+(20) 105(100) 159(68)	1475	0.02 (0.01)	0.31 (0.09)	0.24 (0.05)	0.09 (0.02)	0.14 (0.03)
43	202[M+](30) 159(100) 131(75)	1479	0.41 (0.09)	0.43 (0.09)	0.62 (0.10)	0.42 (0.09)	0.41 (0.09)
44	dehydroaromadendrene	1482	-	0.41 (0.08)	-	-	1.18 (0.18)
45	204[M+](1) 142(100) 141(78)	1495	0.10 (0.02)	0.14 (0.03)	0.01 (*)	0.01 (*)	0.23 (0.04)
46	β-guaiene	1505	0.22 (0.03)	0.01 (*)	0.13 (0.04)	0.15 (0.04)	0.13 (0.03)
47	germacrene D	1510	1.19 (0.18)	3.12 (0.18)	1.63 (0.21)	1.73 (0.18)	2.98 (0.22)
48	ledene	1516	0.02 (0.01)	0.01 (*)	0.01 (*)	0.02 (0.01)	0.13 (0.03)
49	bicyclogermacrene	1521	1.73 (0.18)	7.89 (0.89)	1.38 (0.19)	1.89 (0.18)	8.06 (0.36)
50	220[M+](5) 148(100) 133(95)	1525	0.13 (0.03)	0.37 (0.09)	0.04 (0.01)	0.11 (0.03)	0.19 (0.04)
51	cuparene	1538	0.67 (0.12)	0.78 (0.09)	0.39 (0.09)	0.37 (0.08)	1.71 (0.14)
52	204[M+](22) 93(100) 105(68)	1543	0.04 (0.01)	0.15 (0.03)	0.01(*)	0.04 (0.01)	0.06 (0.01)
53	202[M+](12) 157(100) 142(62)	1554	0.49 (0.09)	0.64 (0.08)	0.29 (0.05)	0.18 (0.02)	0.61 (0.09)
54	204[M+](18) 155(100) 119(82)	1558	0.13 (0.04)	0.01 (*)	0.24 (0.05)	0.33 (0.07)	0.05 (0.01)
55	200[M]+ (72) 143(100) 129(93)	1571	0.53 (0.10)	0.42 (0.09)	0.29 (0.04)	0.35 (0.07)	0.58 (0.09)
56	200[M+](73) 143(100) 157(97)	1579	0.05 (0.01)	0.16 (0.03)	0.01 (*)	0.01 (*)	0.24 (0.05)
57	204[M]+ (6) 69(100) 41(85)	1586	0.19 (0.03)	0.14 (0.02)	0.16 (0.03)	0.08 (0.02)	0.25 (0.05)
58	cadina-3,9-diene	1591	0.13 (0.02)	0.24 (0.04)	0.08 (0.01)	0.07 (0.02)	0.08 (0.01)
59	maaliol	1609	-	-	-	-	-
60	1,4-dimethylazulene	1616	57.13 (1.38)	31.01 (0.96)	55.62 (1.02)	57.32 (1.12)	27.99 (0.86)
61	(+)-spathulenol	1619	-	0.08 (0.02)	-	-	-
62	(-)-globulol	1631	0.52 (0.11)	0.43 (0.10)	0.09 (0.02)	0.13 (0.03)	0.18 (0.04)
63	220[M+](5) 132(100) 43(96)	1637	0.51 (0.10)	0.01 (*)	0.24 (0.05)	0.38 (0.08)	1.21 (0.12)
64	202[M+](3) 166(100) 165(98)	1645	-	0.01 (*)	-	-	-
65	202[M+](2)129(100) 172(72)	1655	1.01 (0.17)	2.18 (0.18)	1.83 (0.16)	1.38 (0.18)	2.64 (0.18)
66	200[M+](2) 145(100) 158(55)	1664	-	0.01 (*)	-	-	-
67	isospathulenol	1667	0.07 (0.02)	0.21 (0.03)	0.16 (0.04)	0.14 (0.03)	0.37 (0.09)
68	218[M+](2) 71(100) 57(93)	1674	-	0.01 (*)	-	-	0.05 (0.01)
69	220[M+](5) 129(100) 144(92)	1678	0.13 (0.03)	0.24 (0.05)	0.15 (0.04)	0.11 (0.02)	0.02 (0.01)
70	220[M+](31) 159(100) 105(81)	1684	0.18 (0.04)	4.32 (0.32)	0.32 (0.08)	0.37 (0.08)	0.52 (0.09)
71	202[M+](92) 143(100) 128(78)	1689	0.21 (0.04)	0.01 (*)	0.19 (0.04)	0.21 (0.04)	0.13 (0.03)
72	220[M+](11) 109(100) 121(51)	1695	-	0.03 (0.01)	-	-	0.04 (0.01)
73	220[M+](13) 159(100) 145(89)	1710	0.07 (0.02)	1.57 (0.21)	0.07 (0.01)	0.10 (0.02)	0.15 (0.03)
74	4-(cyclopent-1-enyl)benzoic acid methyl ester	1722	14.72 (0.67)	16.47 (0.72)	17.01 (0.64)	15.37 (0.46)	11.72 (0.38)
75	216[M+](10) 202(100) 159(96)	1744	0.01 (*)	0.05 (0.01)	0.14 (0.04)	0.01 (*)	0.13 (0.03)
76	216[M+](3) 202(100) 143(81)	1784	0.02 (0.01)	0.01 (*)	0.01 (*)	0.01 (*)	0.04 (0.01)
77	germacra-4(15),5,10(14)-trien-1-alpha-ol	1801	0.61 (0.10)	0.18 (0.03)	0.37 (0.09)	0.52 (0.10)	1.16 (0.12)
78	1,4-dimethyl-7-(1-methylethyl)-azulene	1823	0.04 (0.01)	0.01 (*)	0.08 (0.02)	0.12 (0.02)	0.15 (0.03)
79	methyl-2-methylazulene-1-carboxylate	2025	1.60 (0.12)	1.42 (0.14)	1.43 (0.17)	1.04 (0.14)	2.25 (0.22)
	Total		98.17 (5.59)	97.46 (6.95)	97.97 (5.16)	98.13 (5.36)	97.40 (6.66)
	% Identified		90.75 (4.33)	82.61 (5.18)	89.50 (3.89)	90.14 (3.85)	83.47 (4.82)
	Including:						
	Aliphatics		1.07 (0.17)	1.78 (0.19)	0.84 (0.14)	0.71 (0.14)	0.86 (0.17)
	Aromatics		15.13 (0.73)	16.52 (0.73)	17.40 (0.71)	15.65 (0.53)	11.94 (0.42)
	Monoterpene hydrocarbons		1.03 (0.20)	0.45 (0.09)	0.94 (0.18)	0.78 (0.17)	0.24 (0.05)
	Monoterpenoide hydrocarbons		1.59 (0.11)	0.47 (0.09)	0.85 (0.12)	0.69 (0.09)	1.19 (0.14)
	Sesquiterpene hydrocarbons		69.74 (2.87)	61.25 (3.79)	67.79 (2.51)	71.00 (2.72)	66.44 (3.69)
	Sesquiterpenoide hydrocarbons		2.19 (0.25)	2.14 (0.29)	1.68 (0.23)	1.31 (0.20)	2.80 (0.35)

-, * less than 0.01%. ^a^ Retention index on Quadex 007-5MS column. ^b^ For abbreviations of samples, see Table 1. ( ) standard deviation.

**Table 8:** Volatile compounds detected in the samples C-31–C-35.

No.	Compounds	RI ^a^	Code ^b^				
			C-31	C-32	C-33	C-34	C-35
1	2-methyl-1-propanol	<700	-	0.12 (0.03)	-	-	-
2	pentanal	<700	0.09 (0.02)	0.01 (*)	0.04 (0.01)	0.11 (0.03)	0.02 (0.01)
3	cyclopentanol	748	-	0.09 (0.02)	-	-	-
4	3-methyl-1-butanol	768	0.03 (0.01)	0.51 (0.10)	0.03 (0.01)	0.01 (*)	0.01 (*)
5	hexanal	834	0.02 (0.01)	0.33 (0.09)	0.01 (*)	0.01 (*)	0.01 (*)
6	(*Z*)-3-hexen-1-ol	886	-	0.17 (0.03)	-	-	-
7	1-hexanol	896	-	0.09 (0.02)	-	-	0.08 (0.02)
8	106[M+](58) 91(100) 77(13)	902	0.69 (0.10)	1.35 (0.18)	0.45 (0.09)	0.57 (0.10)	0.38 (0.09)
9	tricyclene	939	-	0.11 (0.03)	-	-	-
10	α-pinene	952	0.02 (0.01)	0.41 (0.09)	0.01 (*)	0.03 (0.01)	0.03 (0.01)
11	camphene	971	-	0.61 (0.14)	-	-	-
12	β-pinene	1004	0.76 (0.11)	0.29 (0.09)	0.49 (0.10)	0.62 (0.12)	0.08 (0.02)
13	3-octanol	1018	-	0.08 (0.01)	-	-	0.01 (*)
14	limonene	1048	0.73 (0.10)	1.97 (0.32)	0.47 (0.09)	0.51 (0.10)	0.13 (0.02)
15	benzene acetaldehyde	1093	0.15 (0.03)	0.03 (0.01)	0.14 (0.03)	0.19 (0.04)	0.08 (0.01)
16	1-octen-3-yl-acetate	1123	0.01 (*)	0.83 (0.10)	0.01 (*)	0.01 (*)	0.51 (0.10)
17	benzene ethanol	1156	0.02 (0.01)	-	0.01 (*)	0.01 (*)	0.01 (*)
18	160[M+](49) 145(100) 117(34)	1224	4.61 (0.28)	2.15 (0.32)	4.23 (0.86)	3.89 (0.72)	3.45 (0.70)
19	β-cyclocitral	1257	1.39 (0.12)	0.33 (0.09)	1.51 (0.18)	1.35 (0.64)	1.16 (0.12)
20	1,2-dihydro-6-methylnaphthalene	1295	0.09 (0.02)	-	0.08 (0.01)	0.02 (0.01)	0.09 (0.02)
21	207[M]+(18) 121(100) 93(71)	1332	0.01 (*)	0.06 (0.01)	0.11 (0.03)	0.08 (0.01)	0.13 (0.13)
22	δ-elemene	1344	0.82 (0.18)	1.25 (0.42)	0.55 (0.09)	0.47 (0.10)	2.28 (0.46)
23	methylnaphthalene	1359	0.17 (0.03)	0.01 (*)	0.07 (0.02)	0.07 (0.02)	0.08 (0.01)
24	202[M+](18) 81(100) 96(83)	1364	0.09 (0.02)	0.37 (0.09)	0.05 (0.01)	0.07 (0.02)	0.21 (0.04)
25	anastreptene	1379	6.69 (0.42)	33.29 (0.86)	7.01 (0.86)	6.92 (0.76)	12.01 (0.98)
26	204[M+](25) 105(100) 161(83)	1382	0.47 (0.09)	-	0.33 (0.08)	0.48 (0.10)	0.35 (0.09)
27	202[M+](5) 159(100) 91(95)	1387	0.19 (0.03)	-	0.03 (0.01)	0.16 (0.03)	0.03 (0.01)
28	202[M+](5) 143(100) 128(92)	1392	1.65 (0.28)	0.51 (0.11)	1.39 (0.12)	1.56 (0.14)	1.11 (0.14)
29	β-elemene	1405	0.13 (0.03)	0.38 (0.09)	0.14 (0.04)	0.38 (0.09)	0.53 (0.12)
30	204[M]+(52) 161(100) 107(89)	1407	-	0.01 (*)	-	-	-
31	202[M]+(2) 143(100) 128(93)	1418	0.34 (0.09)	0.12 (0.03)	0.38 (0.08)	0.20 (0.04)	0.18 (0.03)
32	α-gurjunene	1423	0.15 (0.03)	0.71 (0.18)	0.22 (0.04)	0.19 (0.03)	0.31 (0.08)
33	202[M]+(2) 145(100) 160(35)	1426	0.26 (0.04)	0.01 (*)	0.25 (0.04)	0.35 (0.09)	0.20 (0.03)
34	β-gurjunene	1431	0.23 (0.03)	0.51 (0.10)	0.12 (0.03)	0.15 (0.03)	0.04 (0.01)
35	204[M]+(12) 159(100) 105(95)	1436	0.15 (0.02)	0.20 (0.04)	0.13 (0.03)	0.14 (0.03)	0.01 (*)
36	(-)-aristolene	1439	0.33 (0.09)	0.31 (0.08)	0.29 (0.06)	0.27 (0.04)	0.29 (0.05)
37	γ-gurjunene	1444	0.01 (*)	0.19 (0.04)	0.01 (*)	0.02 (0.01)	0.03 (0.01)
38	γ-maaliene	1449	0.43 (0.09)	0.93 (0.22)	0.82 (0.18)	0.86 (0.11)	0.54 (0.10)
39	α-maaliene	1455	0.29 (0.03)	0.77 (0.18)	0.41 (0.09)	0.39 (0.09)	0.41 (0.09)
40	alloaromadendrene	1459	3.49 (0.24)	9.21 (0.86)	3.21 (0.18)	4.12 (0.26)	4.73 (0.28)
41	selina-5,11-diene	1470	0.62 (0.09)	0.92 (0.12)	0.97 (0.24)	0.91 (0.12)	0.76 (0.14)
42	202[M]+(20) 105(100) 159(68)	1475	0.63 (0.08)	1.73 (0.24)	0.13 (0.02)	0.15 (0.03)	0.21 (0.04)
43	202[M+](30) 159(100) 131(75)	1479	0.54 (0.10)	2.23 (0.18)	0.55 (0.10)	0.61 (0.10)	0.23 (0.04)
44	dehydroaromadendrene	1482	0.39 (0.08)	1.51 (0.18)	0.53 (0.10)	0.65 (0.10)	1.07 (0.18)
45	204[M+](1) 142(100) 141(78)	1495	0.14 (0.03)	0.16 (0.04)	0.08 (0.02)	0.16 (0.03)	0.08 (0.01)
46	β-guaiene	1505	0.02 (0.01)	0.06 (0.01)	0.01 (*)	0.02 (0.01)	0.16 (0.03)
47	germacrene D	1510	2.83 (0.18)	5.11 (0.24)	3.18 (0.32)	3.47 (0.34)	2.76 (0.22)
48	ledene	1516	0.01 (*)	0.04 (0.01)	-	-	0.01 (*)
49	bicyclogermacrene	1521	3.91 (0.28)	9.03 (0.76)	4.28 (0.42)	4.39 (0.44)	9.74 (0.78)
50	220[M+](5) 148(100) 133(95)	1525	0.08 (0.02)	0.04 (0.01)	0.23 (0.03)	0.18 (0.04)	0.49 (0.10)
51	cuparene	1538	0.72 (0.22)	2.03 (0.22)	0.61 (0.11)	0.72 (0.14)	1.52 (0.12)
52	204[M+](22) 93(100) 105(68)	1543	0.11 (0.02)	0.01 (*)	0.05 (0.01)	0.09 (0.02)	0.04 (0.01)
53	202[M+](12) 157(100) 142(62)	1554	0.41 (0.09)	1.22 (0.16)	0.25 (0.03)	0.34 (0.09)	0.52 (0.12)
54	204[M+](18) 155(100) 119(82)	1558	0.03 (0.01)	0.04 (0.01)	0.01 (*)	0.01 (*)	0.01 (*)
55	200[M]+ (72) 143(100) 129(93)	1571	0.26 (0.03)	0.38 (0.09)	0.24 (0.04)	0.25 (0.04)	0.83 (0.12)
56	200[M+](73) 143(100) 157(97)	1579	0.07 (0.02)	0.17 (0.04)	0.03 (0.01)	0.04 (0.01)	0.18 (0.03)
57	204[M]+ (6) 69(100) 41(85)	1586	0.27 (0.06)	0.03 (0.01)	0.12 (0.03)	0.34 (0.09)	0.16 (0.02)
58	cadina-3,9-diene	1591	0.21 (0.05)	0.12 (0.02)	0.15 (0.02)	0.16 (0.03)	0.02 (0.01)
59	maaliol	1609	-	-	-	-	-
60	1,4-dimethylazulene	1616	47.51 (0.96)	9.12 (0.34)	43.12 (0.92)	41.89 (0.88)	27.91 (0.86)
61	(+)-spathulenol	1619	-	0.41 (0.09)	-	-	-
62	(−)-globulol	1631	0.49 (0.18)	0.63 (0.10)	0.25 (0.03)	0.24 (0.03)	0.15 (0.03)
63	220[M+](5) 132(100) 43(96)	1637	0.46 (0.16)	0.33 (0.09)	0.55 (0.11)	0.73 (0.16)	1.08 (0.12)
64	202[M+](3) 166(100) 165(98)	1645	-	-	-	-	-
65	202[M+](2)129(100) 172(72)	1655	1.33 (0.18)	0.74 (0.16)	1.69 (0.16)	1.83 (0.14)	3.03 (0.22)
66	200[M+](2) 145(100) 158(55)	1664	-	-	-	-	-
67	isospathulenol	1667	0.29 (0.09)	0.39 (0.09)	0.41 (0.09)	0.33 (0.09)	0.27 (0.04)
68	218[M+](2) 71(100) 57(93)	1674	0.06 (0.01)	0.38 (0.08)	0.39 (0.09)	0.39 (0.08)	0.01 (*)
69	220[M+](5) 129(100) 144(92)	1678	0.12 (0.02)	0.01 (*)	0.02 (0.01)	0.01 (*)	0.01 (*)
70	220[M+](31) 159(100) 105(81)	1684	0.52 (0.09)	0.13 (0.03)	0.29 (0.05)	0.27 (0.04)	0.49 (0.09)
71	202[M+](92) 143(100) 128(78)	1689	0.09 (0.02)	0.08 (0.02)	0.03 (0.01)	0.03 (0.01)	0.09 (0.01)
72	220[M+](11) 109(100) 121(51)	1695	0.07 (0.01)	0.09 (0.02)	0.09 (0.01)	0.05 (0.01)	0.02 (0.01)
73	220[M+](13) 159(100) 145(89)	1710	0.18 (0.04)	0.23 (0.05)	0.11 (0.03)	0.12 (0.03)	0.26 (0.04)
74	4-(cyclopent-1-enyl)benzoic acid methyl ester	1722	12.38 (0.44)	1.11 (0.28)	16.01 (0.46)	14.89 (0.42)	13.53 (0.44)
75	216[M+](10) 202(100) 159(96)	1744	0.06 (0.01)	0.27 (0.05)	0.02 (0.01)	0.12 (0.03)	0.11 (0.02)
76	216[M+](3) 202(100) 143(81)	1784	0.02 (0.01)	0.01 (*)	0.01 (*)	0.02 (0.01)	0.02 (0.01)
77	germacra-4(15),5,10(14)-trien-1-alpha-ol	1801	0.33 (0.09)	0.91 (0.18)	0.38 (0.09)	0.41 (0.09)	1.01 (0.12)
78	1,4-dimethyl-7-(1-methylethyl)-azulene	1823	0.01 (*)	0.14 (0.03)	0.05 (0.01)	0.03 (0.01)	0.01 (*)
79	methyl-2-methylazulene-1-carboxylate	2025	0.09 (0.02)	0.03 (0.01)	0.12 (0.03)	0.11 (0.03)	1.28 (0.12)
	Total		99.77 (6.26)	98.16 (8.94)	97.96 (6.98)	98.17 (7.46)	97.59 (7.78)
	% Identified		85.86 (4.30)	85.10 (6.88)	85.72 (4.86)	84.93 (5.22)	83.67 (5.61)
	Including:						
	Aliphatics		0.15 (0.04)	2.23 (0.40)	0.09 (0.02)	0.14 (0.03)	0.64 (0.13)
	Aromatics		12.81 (0.53)	1.15 (0.29)	16.31 (0.52)	15.18 (0.49)	13.79 (0.48)
	Monoterpene hydrocarbons		1.51 (0.22)	3.39 (0.67)	0.97 (0.19)	1.16 (0.23)	0.24 (0.05)
	Monoterpenoide hydrocarbons		1.39 (0.12)	0.33 (0.09)	1.51 (0.18)	1.35 (0.64)	1.16 (0.12)
	Sesquiterpene hydrocarbons		69.13 (3.10)	76.54 (5.14)	66.06 (3.80)	66.42 (3.68)	66.14 (4.64)
	Sesquiterpenoide hydrocarbons		0.87 (0.29)	1.46 (0.29)	0.78 (0.15)	0.68 (0.15)	1.70 (0.19)

-, * less than 0.01%. ^a^ Retention index on Quadex 007-5MS column. ^b^ For abbreviations of samples, see Table 1. ( ) standard deviation.

**Table 9:** Volatile compounds detected in the samples C-36–C-40.

No.	Compounds	RI ^a^	Code ^b^				
			C-36	C-37	C-38	C-39	C-40
1	2-methyl-1-propanol	<700	0.05 (0.01)	-	-	-	0.01 (*)
2	pentanal	<700	0.01 (*)	0.01 (*)	0.02 (0.01)	0.11 (0.02)	0.01 (*)
3	cyclopentanol	748	0.03 (0.01)	-	-	-	0.01 (*)
4	3-methyl-1-butanol	768	0.25 (0.04)	0.02 (0.01)	0.01 (*)	0.01 (*)	0.01 (*)
5	hexanal	834	0.15 (0.03)	0.02 (0.01)	0.01 (*)	0.01 (*)	0.01 (*)
6	(*Z*)-3-hexen-1-ol	886	0.09 (0.01)	-	-	-	-
7	1-hexanol	896	0.14 (0.03)	0.08 (0.01)	0.05 (0.01)	0.29 (0.03)	0.22 (0.02)
8	106[M+](58) 91(100) 77(13)	902	0.57 (0.12)	0.31 (0.08)	0.30 (0.08)	0.04 (0.01)	-
9	tricyclene	939	0.03 (0.01)	-	-	-	0.01 (*)
10	α-pinene	952	0.21 (0.04)	0.06 (0.01)	0.15 (0.03)	0.03 (0.01)	0.01 (*)
11	camphene	971	0.53 (0.12)	-	-	-	0.01 (*)
12	β-pinene	1004	0.16 (0.03)	0.01 (*)	0.21 (0.03)	0.39 (0.09)	0.41 (0.09)
13	3-octanol	1018	0.25 (0.04)	0.01 (*)	0.01 (*)	0.05 (0.01)	0.08 (0.01)
14	limonene	1048	0.32 (0.06)	0.16 (0.02)	0.07 (0.01)	0.51 (0.09)	0.04 (0.01)
15	benzene acetaldehyde	1093	0.02 (0.01)	0.25 (0.03)	0.13 (0.02)	0.04 (0.01)	0.01 (*)
16	1-octen-3-yl-acetate	1123	1.27 (0.12)	0.31 (0.06)	0.29 (0.03)	0.59 (0.10)	1.52 (0.10)
17	benzene ethanol	1156	-	0.01 (*)	0.01 (*)	0.01 (*)	-
18	160[M+](49) 145(100) 117(34)	1224	2.27 (0.32)	3.51 (0.14)	3.01 (0.14)	1.31 (0.11)	2.23 (0.12)
19	β-cyclocitral	1257	0.59 (0.10)	0.73 (0.10)	1.31 (0.10)	1.59 (0.10)	0.47 (0.09)
20	1,2-dihydro-6-methylnaphthalene	1295	-	0.17 (0.03)	0.04 (0.01)	0.11 (0.03)	-
21	207[M]+(18) 121(100) 93(71)	1332	0.05 (0.01)	0.13 (0.02)	0.18 (0.04)	0.03 (0.01)	0.06 (0.01)
22	δ-elemene	1344	1.27 (0.12)	2.11 (0.12)	2.75 (0.14)	0.27 (0.04)	1.41 (0.10)
23	methylnaphthalene	1359	0.01 (*)	0.03 (0.01)	0.04 (0.01)	0.11 (0.03)	0.01 (*)
24	202[M+](18) 81(100) 96(83)	1364	0.11 (0.03)	0.21 (0.05)	0.23 (0.03)	0.11 (0.03)	0.05 (0.01)
25	anastreptene	1379	21.18 (0.92)	10.71 (0.42)	11.03 (0.45)	4.96 (0.14)	8.95 (0.42)
26	204[M+](25) 105(100) 161(83)	1382	0.04 (0.01)	0.18 (0.03)	0.63 (0.09)	0.07 (0.01)	0.04 (0.01)
27	202[M+](5) 159(100) 91(95)	1387	-	0.02 (0.01)	0.04 (0.01)	0.09 (0.01)	0.01 (*)
28	202[M+](5) 143(100) 128(92)	1392	0.57 (0.12)	1.11 (0.10)	1.31 (0.10)	0.96 (0.10)	0.67 (0.10)
29	β-elemene	1405	0.37 (0.09)	0.31 (0.08)	0.45 (0.09)	0.18 (0.03)	0.41 (0.09)
30	204[M]+(52) 161(100) 107(89)	1407	0.02 (0.01)	-	-	-	0.11 (0.03)
31	202[M]+(2) 143(100) 128(93)	1418	0.10 (0.02)	0.29 (0.07)	0.31 (0.07)	0.08 (0.01)	0.01 (*)
32	α-gurjunene	1423	0.31 (0.07)	0.27 (0.04)	0.23 (0.04)	0.21 (0.03)	0.15 (0.02)
33	202[M]+(2) 145(100) 160(35)	1426	0.13 (0.02)	0.48 (0.09)	0.33 (0.06)	0.09 (0.01)	0.29 (0.04)
34	β-gurjunene	1431	0.04 (0.01)	0.01 (*)	0.01 (*)	0.01 (*)	0.09 (0.01)
35	204[M]+(12) 159(100) 105(95)	1436	0.28 (0.04)	0.01 (*)	0.13 (0.03)	0.01 (*)	0.31 (0.08)
36	(−)-aristolene	1439	0.11 (0.03)	0.55 (0.12)	0.29 (0.06)	0.09 (0.01)	0.01 (*)
37	γ-gurjunene	1444	0.11 (0.02)	0.01 (*)	0.01 (*)	0.34 (0.08)	0.01 (*)
38	γ-maaliene	1449	0.69 (0.11)	1.12 (0.14)	0.95 (0.10)	0.16 (0.03)	0.59 (0.10)
39	α-maaliene	1455	0.61 (0.10)	0.31 (0.08)	0.35 (0.08)	0.29 (0.04)	0.55 (0.09)
40	alloaromadendrene	1459	6.25 (0.32)	3.99 (0.22)	5.03 (0.28)	1.22 (0.10)	5.07 (0.44)
41	selina-5,11-diene	1470	0.26 (0.26)	1.23 (0.12)	0.99 (0.10)	0.21 (0.04)	0.67 (0.10)
42	202[M]+(20) 105(100) 159(68)	1475	0.91 (0.12)	0.31 (0.09)	0.18 (0.03)	0.01 (*)	0.44 (0.09)
43	202[M+](30) 159(100) 131(75)	1479	1.04 (0.11)	0.35 (0.08)	0.19 (0.03)	0.43 (0.09)	0.53 (0.10)
44	dehydroaromadendrene	1482	0.91 (0.12)	0.99 (0.10)	1.36 (0.10)	-	0.47 (0.09)
45	204[M+](1) 142(100) 141(78)	1495	0.33 (0.06)	0.21 (0.04)	0.04 (0.01)	0.11 (0.03)	0.16 (0.03)
46	β-guaiene	1505	0.17 (0.02)	0.05 (0.01)	0.12 (0.03)	0.24 (0.03)	0.01 (*)
47	germacrene D	1510	3.17 (0.18)	2.63 (0.20)	2.56 (0.20)	1.05 (0.10)	3.03 (0.22)
48	ledene	1516	0.05 (0.01)	0.01 (*)	0.01 (*)	0.01 (*)	0.01 (*)
49	bicyclogermacrene	1521	15.63 (0.22)	11.01 (0.22)	10.37 (0.22)	1.73 (0.10)	8.83 (0.20)
50	220[M+](5) 148(100) 133(95)	1525	0.38 (0.06)	0.35 (0.07)	0.39 (0.09)	0.11 (0.02)	0.34 (0.09)
51	cuparene	1538	1.06 (0.12)	0.95 (0.10)	1.33 (0.10)	0.64 (0.09)	0.68 (0.10)
52	204[M+](22) 93(100) 105(68)	1543	0.04 (0.01)	0.11 (0.03)	0.15 (0.03)	0.01 (*)	0.07 (0.01)
53	202[M+](12) 157(100) 142(62)	1554	0.18 (0.03)	0.45 (0.09)	0.29 (0.04)	0.47 (0.09)	0.50 (0.09)
54	204[M+](18) 155(100) 119(82)	1558	0.02 (0.01)	0.11 (0.02)	0.01 (*)	0.19 (0.02)	0.01 (*)
55	200[M]+ (72) 143(100) 129(93)	1571	0.41 (0.08)	0.41 (0.08)	0.43 (0.09)	0.55 (0.09)	0.42 (0.09)
56	200[M+](73) 143(100) 157(97)	1579	0.16 (0.03)	0.13 (0.03)	0.21 (0.03)	0.02 (0.01)	0.08 (0.01)
57	204[M]+ (6) 69(100) 41(85)	1586	0.09 (0.02)	0.41 (0.09)	0.15 (0.02)	0.22 (0.03)	0.10 (0.02)
58	cadina-3,9-diene	1591	0.23 (0.04)	0.07 (0.01)	0.22 (0.04)	0.09 (0.01)	0.23 (0.03)
59	maaliol	1609	-	-	-	-	-
60	1,4-dimethylazulene	1616	20.08 (0.76)	27.07 (0.78)	26.27 (0.76)	59.23 (0.92)	30.48 (0.64)
61	(+)-spathulenol	1619	0.31 (0.09)	-	-	-	0.09 (0.01)
62	(−)-globulol	1631	0.11 (0.02)	0.21 (0.03)	0.18 (0.04)	0.37 (0.09)	0.61 (0.10)
63	220[M+](5) 132(100) 43(96)	1637	0.19 (0.03)	0.59 (0.10)	0.82 (0.12)	0.55 (0.10)	0.01 (*)
64	202[M+](3) 166(100) 165(98)	1645	0.01 (*)	-	-	-	0.01 (*)
65	202[M+](2)129(100) 172(72)	1655	1.18 (0.12)	3.65 (0.22)	3.08 (0.24)	0.97 (0.10)	2.05 (0.18)
66	200[M+](2) 145(100) 158(55)	1664	0.03 (0.01)	-	-	-	0.02 (0.01)
67	isospathulenol	1667	0.79 (0.10)	0.41 (0.09)	0.16 (0.03)	0.19 (0.03)	0.27 (0.03)
68	218[M+](2) 71(100) 57(93)	1674	0.21 (0.03)	0.01 (*)	0.13 (0.03)	-	0.01 (*)
69	220[M+](5) 129(100) 144(92)	1678	-	0.01 (*)	0.01 (*)	0.09 (0.02)	0.27 (0.03)
70	220[M+](31) 159(100) 105(81)	1684	0.79 (0.10)	0.43 (0.09)	0.41 (0.14)	0.22 (0.03)	4.35 (0.36)
71	202[M+](92) 143(100) 128(78)	1689	0.01 (*)	0.15 (0.02)	0.09 (0.02)	0.22 (0.03)	0.01 (*)
72	220[M+](11) 109(100) 121(51)	1695	0.11 (0.02)	0.03 (0.01)	0.12 (0.03)	-	0.03 (0.01)
73	220[M+](13) 159(100) 145(89)	1710	1.03 (0.12)	0.21 (0.03)	0.17 (0.03)	0.13 (0.02)	1.53 (0.10)
74	4-(cyclopent-1-enyl)benzoic acid methyl ester	1722	8.11 (0.32)	15.93 (0.34)	14.47 (0.42)	14.67 (0.40)	15.97 (0.42)
75	216[M+](10) 202(100) 159(96)	1744	0.08 (0.01)	0.12 (0.03)	0.01 (*)	0.01 (*)	0.03 (0.01)
76	216[M+](3) 202(100) 143(81)	1784	0.01 (*)	0.03 (0.01)	0.01 (*)	0.01 (*)	0.01 *
77	germacra-4(15),5,10(14)-trien-1-alpha-ol	1801	0.28 (0.03)	0.79 (0.12)	0.69 (0.10)	0.63 (0.10)	0.29 (0.04)
78	1,4-dimethyl-7-(1-methylethyl)-azulene	1823	0.60 (0.18)	0.02 (0.01)	0.01 (*)	0.01 (*)	0.01 (*)
79	methyl-2-methylazulene-1-carboxylate	2025	0.91 (0.11)	1.31 (0.10)	1.54 (0.11)	1.42 (0.10)	1.59 (0.12)
	Total		99.07 (6.68)	98.26 (5.66)	97.09 (5.58)	99.18 (4.35)	98.07 (5.30)
	% Identified		87.72 (5.01)	83.94 (3.94)	83.73 (3.95)	92.07 (3.37)	83.32 (3.67)
	Including:						
	Aliphatics		2.24 (0.29)	0.45 (0.09)	0.39 (0.05)	1.06 (0.16)	1.87 (0.13)
	Aromatics		8.14 (0.33)	16.39 (0.41)	14.69 (0.46)	14.94 (0.47)	15.99 (0.42)
	Monoterpene hydrocarbons		1.25 (0.26)	0.23 (0.03)	0.43 (0.07)	0.93 (0.19)	0.48 (0.10)
	Monoterpenoide hydrocarbons		0.59 (0.10)	0.73 (0.10)	1.31 (0.10)	1.59 (0.10)	0.47 (0.09)
	Sesquiterpene hydrocarbons		73.38 (3.71)	64.21 (3.09)	65.03 (3.09)	71.57 (2.23)	61.95 (2.67)
	Sesquiterpenoide hydrocarbons		2.12 (0.32)	1.93 (0.22)	1.88 (0.18)	1.98 (0.22)	2.56 (0.26)

-, * less than 0.01%. ^a^ Retention index on Quadex 007-5MS column. ^b^ For abbreviations of samples, see Table 1. ( ) standard deviation.

**Table 10:** Volatile compounds detected in the samples C-41–C-43.

No.	Compounds	RI ^a^	Code ^b^		
			C-41	C-42	C-43
1	2-methyl-1-propanol	<700	-	-	-
2	pentanal	<700	0.09 (0.01)	0.03 (0.01)	0.02 (0.01)
3	cyclopentanol	748	-	-	-
4	3-methyl-1-butanol	768	0.01 (*)	0.01 (*)	0.04 (0.01)
5	hexanal	834	0.01 (*)	0.01 (*)	0.01 (*)
6	(*Z*)-3-hexen-1-ol	886	-	-	-
7	1-hexanol	896	0.33 (0.08)	0.45 (0.09)	0.09 (0.01)
8	106[M+](58) 91(100) 77(13)	902	0.15 (0.03)	0.04 (0.01)	0.47 (0.09)
9	tricyclene	939	-	-	-
10	α-pinene	952	0.13 (0.02)	0.15 (0.03)	0.05 (0.01)
11	camphene	971	-	-	-
12	β-pinene	1004	0.40 (0.09)	0.35 (0.08)	0.03 (0.01)
13	3-octanol	1018	0.01 (*)	0.01 (*)	0.02 (0.01)
14	limonene	1048	0.37 (0.08)	0.05 (0.01)	0.16 (0.03)
15	benzene acetaldehyde	1093	0.21 (0.03)	0.05 (0.01)	0.06 (0.01)
16	1-octen-3-yl-acetate	1123	0.52 (0.10)	0.27 (0.04)	0.58 (0.09)
17	benzene ethanol	1156	0.01 (*)	0.01 (*)	0.02 (0.01)
18	160[M+](49) 145(100) 117(34)	1224	1.53 (0.11)	2.03 (0.18)	3.54 (0.32)
19	β-cyclocitral	1257	0.82 (0.10)	0.69 (0.10)	1.15 (0.11)
20	1,2-dihydro-6-methylnaphthalene	1295	0.14 (0.03)	0.23 (0.04)	0.09 (0.01)
21	207[M]+(18) 121(100) 93(71)	1332	0.10 (0.02)	0.02 (0.01)	0.15 (0.03)
22	δ-elemene	1344	0.24 (0.03)	0.18 (0.03)	2.18 (0.20)
23	methylnaphthalene	1359	0.02 (0.01)	0.02 (0.01)	0.05 (0.01)
24	202[M+](18) 81(100) 96(83)	1364	0.11 (0.02)	0.07 (0.01)	0.22 (0.04)
25	anastreptene	1379	5.28 (0.26)	4.89 (0.22)	12.67 (0.68)
26	204[M+](25) 105(100) 161(83)	1382	0.06 (0.01)	0.10 (0.02)	0.33 (0.07)
27	202[M+](5) 159(100) 91(95)	1387	0.23 (0.03)	0.22 (0.03)	0.03 (0.01)
28	202[M+](5) 143(100) 128(92)	1392	1.03 (0.10)	1.23 (0.10)	1.31 (0.11)
29	β-elemene	1405	0.62 (0.09)	0.18 (0.03)	0.47 (0.09)
30	204[M]+(52) 161(100) 107(89)	1407	-	-	-
31	202[M]+(2) 143(100) 128(93)	1418	0.09 (0.01)	0.11 (0.02)	0.17 (0.03)
32	α-gurjunene	1423	0.08 (0.01)	0.10 (0.02)	0.18 (0.03)
33	202[M]+(2) 145(100) 160(35)	1426	0.17 (0.03)	0.34 (0.07)	0.21 (0.04)
34	β-gurjunene	1431	0.01 (*)	0.01 (*)	0.04 (0.01)
35	204[M]+(12) 159(100) 105(95)	1436	0.01 (*)	0.02 (0.01)	0.03 (0.01)
36	(−)-aristolene	1439	0.03 (0.01)	0.03 (0.01)	0.36 (0.09)
37	γ-gurjunene	1444	0.27 (0.03)	0.41 (0.09)	0.06 (0.01)
38	γ-maaliene	1449	0.29 (0.04)	0.18 (0.03)	0.55 (0.09)
39	α-maaliene	1455	0.27 (0.03)	0.23 (0.04)	0.41 (0.09)
40	alloaromadendrene	1459	1.12 (0.10)	2.07 (0.22)	4.72 (0.38)
41	selina-5,11-diene	1470	0.18 (0.03)	0.39 (0.09)	0.85 (0.11)
42	202[M]+(20) 105(100) 159(68)	1475	0.08 (0.01)	0.08 (0.01)	0.18 (0.03)
43	202[M+](30) 159(100) 131(75)	1479	0.51 (0.02)	0.62 (0.10)	0.33 (0.08)
44	dehydroaromadendrene	1482	-	-	1.05 (0.11)
45	204[M+](1) 142(100) 141(78)	1495	0.02 (0.01)	0.01 (*)	0.09 (0.01)
46	β-guaiene	1505	0.08 (0.01)	0.08 (0.01)	0.13 (0.03)
47	germacrene D	1510	1.63 (0.10)	1.75 (0.10)	2.89 (0.24)
48	ledene	1516	0.01 (*)	0.01 (*)	0.05 (0.01)
49	bicyclogermacrene	1521	1.40 (0.10)	1.68 (0.10)	10.22 (0.38)
50	220[M+](5) 148(100) 133(95)	1525	0.02 (0.01)	0.07 (0.01)	0.47 (0.09)
51	cuparene	1538	0.29 (0.04)	0.38 (0.03)	1.56 (0.12)
52	204[M+](22) 93(100) 105(68)	1543	0.01 (*)	0.02 (0.01)	0.02 (0.01)
53	202[M+](12) 157(100) 142(62)	1554	0.36 (0.08)	0.24 (0.03)	0.51 (0.09)
54	204[M+](18) 155(100) 119(82)	1558	0.18 (0.03)	0.22 (0.04)	0.05 (0.01)
55	200[M]+ (72) 143(100) 129(93)	1571	0.29 (0.04)	0.29 (0.03)	0.83 (0.11)
56	200[M+](73) 143(100) 157(97)	1579	0.02 (0.01)	0.01 (*)	0.18 (0.03)
57	204[M]+ (6) 69(100) 41(85)	1586	0.08 (0.01)	0.08 (0.01)	0.20 (0.04)
58	cadina-3,9-diene	1591	0.08 (0.01)	0.02 (0.01)	0.06 (0.01)
59	maaliol	1609	-	-	-
60	1,4-dimethylazulene	1616	54.71 (0.88)	56.82 (0.86)	27.11 (0.68)
61	(+)-spathulenol	1619	-	-	-
62	(−)-globulol	1631	0.11 (0.02)	0.17 (0.04)	0.16 (0.03)
63	220[M+](5) 132(100) 43(96)	1637	0.32 (0.08)	0.38 (0.08)	1.11 (0.10)
64	202[M+](3) 166(100) 165(98)	1645	-	-	-
65	202[M+](2)129(100) 172(72)	1655	1.87 (0.12)	1.38 (0.10)	2.87 (0.18)
66	200[M+](2) 145(100) 158(55)	1664	-	-	-
67	isospathulenol	1667	0.34 (0.08)	0.22 (0.03)	0.34 (0.07)
68	218[M+](2) 71(100) 57(93)	1674	-	-	0.05 (0.01)
69	220[M+](5) 129(100) 144(92)	1678	0.11 (0.03)	0.11 (0.02)	0.06 (0.01)
70	220[M+](31) 159(100) 105(81)	1684	0.34 (0.08)	0.33 (0.08)	0.47 (0.09)
71	202[M+](92) 143(100) 128(78)	1689	0.15 (0.02)	0.14 (0.03)	0.12 (0.02)
72	220[M+](11) 109(100) 121(51)	1695	-	-	0.04 (0.01)
73	220[M+](13) 159(100) 145(89)	1710	0.15 (0.02)	0.07 (0.01)	0.21 (0.03)
74	4-(cyclopent-1-enyl)benzoic acid methyl ester	1722	17.31 (0.44)	15.02 (0.42)	12.59 (0.42)
75	216[M+](10) 202(100) 159(96)	1744	0.07 (0.01)	0.10 (0.02)	0.09 (0.02)
76	216[M+](3) 202(100) 143(81)	1784	0.05 (0.01)	0.01 (*)	0.04 (0.01)
77	germacra-4(15),5,10(14)-trien-1-alpha-ol	1801	0.47 (0.09)	0.51 (0.09)	1.02 (0.10)
78	1,4-dimethyl-7-(1-methylethyl)-azulene	1823	0.08 (0.01)	0.09 (0.01)	0.06 (0.01)
79	methyl-2-methylazulene-1-carboxylate	2025	1.42 (0.10)	1.32 (0.10)	1.54 (0.14)
	Total		97.50 (4.35)	97.41 (4.35)	98.02 (6.19)
	% Identified		89.39 (3.40)	89.07 (3.31)	83.64 (4.46)
	Including:				
	Aliphatics		0.97 (0.19)	0.78 (0.14)	0.76 (0.13)
	Aromatics		17.69 (0.51)	15.33 (0.48)	12.81 (0.46)
	Monoterpene hydrocarbons		0.90 (0.19)	0.55 (0.12)	0.24 (0.05)
	Monoterpenoide hydrocarbons		0.82 (0.10)	0.69 (0.10)	1.15 (0.11)
	Sesquiterpene hydrocarbons		67.14 (2.21)	70.01 (2.30)	66.64 (3.47)
	Sesquiterpenoide hydrocarbons		1.87 (0.20)	1.71 (0.17)	2.04 (0.24)

-, * less than 0.01%. ^a^ Retention index on Quadex 007-5MS column. ^b^ For abbreviations of samples, see Table 1. ( ) standard deviation.

## Data Availability

Not applicable.

## References

[1] Saritas Y., Mekem Sonwa M., Iznaguen H., König W.A., Muhle H., Mues R. (2001). Volatile Constituents in Mosses (Musci). Phytochemistry.

[2] Linde J., Combrinck S., van Vuuren S., van Rooy J., Ludwiczuk A., Mokgalaka N. (2016). Volatile Constituents and Antimicrobial Activities of Nine South African Liverwort Species. Phytochem. Lett..

[3] Nagashima F., Asakawa Y. (2011). Terpenoids and Bibenzyls from Three Argentine Liverworts. Molecules.

[4] Zhu M.Z., Li Y., Zhou J.C., Lu J.H., Zhu R.X., Qiao Y.N., Zhang J.Z., Zong Y., Wang X., Jin X.Y. (2020). Terpenoids from the Chinese Liverwort *Odontoschisma grosseverrucosum* and Their Antifungal Virulence Activity. Phytochemistry.

[5] Veljić M., Ćirić A., Soković M., Janaćković P., Marin P.D. (2010). Antibacterial and Antifungal Activity of the Liverwort (*Ptilidium pulcherrimum*) Methanol Extract. Arch. Biol. Sci..

[6] Söderström L., Váňa J., Crandall-Stotler B., Renner M.A.M., Hagborg A., von Konrat M. (2015). Notes on Early Land Plants Today. 68. Miscellaneous Notes on *Marchantiophyta*. Phytotaxa.

[7] Fan H., Wei G., Chen X., Guo H., Crandall-Stotler B., Köllner T.G., Chen F. (2021). Sesquiterpene Biosynthesis in a Leafy Liverwort *Radula lindenbergiana* Gottsche Ex C. Hartm. Phytochemistry.

[8] Zhao J., Davis L.C., Verpoorte R. (2005). Elicitor Signal Transduction Leading to Production of Plant Secondary Metabolites. Biotechnol. Adv..

[9] Lunić T.M., Mandić M.R., Oalđe Pavlović M.M., Sabovljević A.D., Sabovljević M.S., Božić Nedeljković B., Božić B. (2022). The Influence of Seasonality on Secondary Metabolite Profiles and Neuroprotective Activities of Moss *Hypnum cupressiforme* Extracts: In Vitro and in Silico Study. Plants.

[10] Schuster R.M. (1969). Hepaticae and Anthocerotae of North America East of the Hundredth Meridian.

[11] Buczkowska K., Bączkiewicz A. (2011). New Taxon of the Genus *Calypogeia* (*Jungermanniales*, *Hepaticae*) in Poland. Acta Soc. Bot. Pol..

[12] Schuster R.M. (1995). Phylogenetic and Taxonomic Studies of *Jungermanniidae*, III *Calypogeiaceae*. Fragm. Florist. Geobot..

[13] Grolle R., Long D. (2000). An Annotated Check-List of the *Hepaticae* and *Anthocerotae* of Europe and Macaronesia. J. Bryol..

[14] Buczkowska K. (2004). The Genus *Calypogeia raddi* (*Jungermanniales*, *Hepaticae*) in Poland, Biometrical Analysis of Morphological and Anatomical Variation. Nova Hedwig..

[15] Buczkowska K., Bakalin V., Bczkiewicz A., Aguero B., Gonera P., Elipiko M., Szczeciñska M., Sawicki J. (2018). Does *Calypogeia azurea* (*Calypogeiaceae*, *Marchantiophyta*) Occur Outside Europe? Molecular and Morphological Evidence. PLoS ONE.

[16] Ludwiczuk A., Asakawa Y. (2014). Fingerprinting of Secondary Metabolites of Liverworts: Chemosystematic Approach. J. AOAC Int..

[17] Shic Hong W. (1990). The Family Calypogeiaceae in North America West of the Hundredth Meridian.

[18] Gordon M. (1952). The Azulenes. Chem. Rev..

[19] Warmers U., Wihstutz K., Bülow N., Low È., Fricke C., König W.A., Nig È. (1998). Sesquiterpene Constituents of the Liverwort Calypogeia Muelleriana. Phytochemistry.

[20] Glime J. (2021). Aquatic and Wet *Marchantiophyta*, Order *Jungermanniales*: *Jungermanniineae*. Bryophyte Ecology.

[21] Sasidharan S., Chen Y., Saravanan D., Sundram K.M., Yoga Latha L. (2011). Extraction, Isolation and Characterization of Bioactive Compounds from Plants’ Extracts. Afr. J. Tradit. Complementary Altern. Med..

[22] Fabricant D.S., Farnsworth N.F. (2001). The Value of Plants Used in Traditional Medicine for Drug Discovery. Environ. Health Perspect..

[23] Handley A.J. (1999). Extraction Methods in Organic Analysis.

[24] Smith R.M. (2003). Before the Injection—Modern Methods of Sample Preparation for Separation Techniques. J. Chromatogr. A.

[25] Rowan D.D. (2011). Volatile Metabolites. Metabolites.

[26] Nogués S., Allen D.J., Morison J.I.L., Baker N.R. (1998). Ultraviolet-B Radiation Effects on Water Relations, Leaf Development, and Photosynthesis in Droughted Pea Plants. Plant Physiol..

[27] Carvalho R.F., Takaki M., Azevedo R.A. (2011). Plant Pigments: The Many Faces of Light Perception. Acta Physiol. Plant..

[28] Zoratti L., Karppinen K., Escobar A.L., Häggman H., Jaakola L. (2014). Light-Controlled Flavonoid Biosynthesis in Fruits. Front. Plant Sci..

[29] Yang L., Wen K.S., Ruan X., Zhao Y.X., Wei F., Wang Q. (2018). Response of Plant Secondary Metabolites to Environmental Factors. Molecules.

[30] Wawrzyniak R., Wasiak W., Ba̧czkiewicz A., Buczkowska K. (2014). Volatile Compounds in Cryptic Species of the *Aneura pinguis* Complex and *Aneura maxima* (*Marchantiophyta*, *Metzgeriidae*). Phytochemistry.

[31] Wawrzyniak R., Wasiak W., Jasiewicz B., Bączkiewicz A., Buczkowska K. (2021). Chemical Fingerprinting of Cryptic Species and Genetic Lineages of *Aneura pinguis* (L.) Dumort. (*Marchantiophyta*, *Metzgeriidae*). Molecules.

[32] Siegel U., Mues R., Dönig R., Eicher T.H., Blechschmidt M., Beckeri H. (1992). Ten Azulenes from *Plagiochila longispina* and *Calypogeia azurea*. Phytochemistry.

[33] Nakagawara S., Katoh K., Kusumi T., Komura H., Nomoto K., Konno H., Huneck S., Takeda R. (1992). Two Azulenes Produced by the Liverwort, *Calypogeia azurea*, during in Vitro Culture. Phytochemistry.

